# Supplementary material for: Comparison of serum vitamin D level and vitamin D receptor gene FokI polymorphism in leprosy patients with and without trophic ulcers: A case-control study
Source: PLoS Negl Trop Dis. 2026 Apr 10;20(4):e0014205. doi: 10.1371/journal.pntd.0014205 (PMC13086432; doi:10.1371/journal.pntd.0014205)
Supplement: S1 File — Strengthening the Reporting of Observational Studies in Epidemiology (STROBE) checklist for the manuscript. (PDF) [file pntd.0014205.s002.pdf]

STROBE Statement—Checklist of items that should be included in reports of ***case-control studies***
Study Title: Comparison of serum vitamin D level and vitamin D receptor gene *FokI* polymorphism in leprosy patients with and without trophic ulcers: A case-control study

	Item No	Recommendation
**Title and abstract**	1	(*a*) Indicate the study's design with a commonly used term in the title or the abstract<br>**Location in manuscript: Study title in Line 1-2**
		(*b*) Provide in the abstract an informative and balanced summary of what was done and what was found<br>**Location in manuscript: Abstract in Line 16-39**
**Introduction**		
Background/rationale	2	Explain the scientific background and rationale for the investigation being reported<br>**Location in manuscript: Introduction in Lines 49-75**
Objectives	3	State specific objectives, including any prespecified hypotheses<br>**Location in manuscript: Introduction in Lines 75-77**
**Methods**		
Study design	4	Present key elements of study design early in the paper<br>**Location in manuscript: Introduction in Lines 85-89**
Setting	5	Describe the setting, locations, and relevant dates, including periods of recruitment, exposure, follow-up, and data collection<br>**Location in manuscript: Introduction in Lines 90-127**
Participants	6	(*a*) Give the eligibility criteria, and the sources and methods of case ascertainment and control selection. Give the rationale for the choice of cases and controls<br>**Location in manuscript: Elaborated in the published protocol**<br>**Halim PA, Sirait SP, Miranda E, Ariani Y, Indriatmi W, Thio HB. Comparison of serum vitamin D level and vitamin D receptor gene FokI polymorphism in leprosy patients with and without trophic ulcers: protocol for a case-control study. F1000Res. 2025;14: 57. doi:10.12688/f1000research.157869.3**
		(*b*) For matched studies, give matching criteria and the number of controls per case<br>**Not applicable**
Variables	7	Clearly define all outcomes, exposures, predictors, potential confounders, and effect modifiers. Give diagnostic criteria, if applicable<br>**Location in manuscript: Elaborated in the cited published protocol**
Data sources/ measurement	8*	For each variable of interest, give sources of data and details of methods of assessment (measurement). Describe comparability of assessment methods if there is more than one group<br>**Location in manuscript: Elaborated in the cited published protocol**
Bias	9	Describe any efforts to address potential sources of bias<br>**Location in manuscript: Elaborated in the cited published protocol**
Study size	10	Explain how the study size was arrived at<br>**Location in manuscript: Elaborated in the cited published protocol**
Quantitative variables	11	Explain how quantitative variables were handled in the analyses. If applicable, describe which groupings were chosen and why<br>**Location in manuscript: Line 116-119 for vitamin D level categorization, elaborated in the cited published protocol**
Statistical methods	12	(*a*) Describe all statistical methods, including those used to control for confounding<br>**Location in manuscript: Statistical analysis in Lines 134-139, more detailed**

		**analysis description is in the cited published protocol**
		(*b*) Describe any methods used to examine subgroups and interactions<br>**Location in manuscript: Statistical analysis in Lines 134-139, more detailed analysis description is in the cited published protocol**
		(*c*) Explain how missing data were addressed<br>**Not applicable, as there were no missing data.**
		(*d*) If applicable, explain how matching of cases and controls was addressed<br>**Not applicable**
		(*e*) Describe any sensitivity analyses<br>**Not performed**
**Results**		
Participants	13*	(a) Report numbers of individuals at each stage of study—eg numbers potentially eligible, examined for eligibility, confirmed eligible, included in the study, completing follow-up, and analysed<br>**Location in manuscript: Figure 2**
		(b) Give reasons for non-participation at each stage<br>**Location in manuscript: Figure 2**
		(c) Consider use of a flow diagram<br>**Location in manuscript: Figure 2**
Descriptive data	14*	(a) Give characteristics of study participants (eg demographic, clinical, social) and information on exposures and potential confounders<br>**Location in manuscript: Tables 1 and 2**
		(b) Indicate number of participants with missing data for each variable of interest<br>**No missing data**
Outcome data	15*	Report numbers in each exposure category, or summary measures of exposure<br>**Location in manuscript: Tables 4**
Main results	16	(*a*) Give unadjusted estimates and, if applicable, confounder-adjusted estimates and their precision (eg, 95% confidence interval). Make clear which confounders were adjusted for and why they were included<br>**Location in manuscript: Line 194-203 and Table 5**
		(*b*) Report category boundaries when continuous variables were categorized<br>**Location in manuscript: Table 4, as defined in Line 116-119 for vitamin D level categorization**
		(*c*) If relevant, consider translating estimates of relative risk into absolute risk for a meaningful time period<br>**Not applicable.**

Other analyses	17	Report other analyses done—eg analyses of subgroups and interactions, and sensitivity analyses<br>**Location in manuscript: Genetic model analysis Line 213-220 and Table 6.**
**Discussion**		
Key results	18	Summarise key results with reference to study objectives<br>**Location in manuscript: Discussion in Line 223-229**
Limitations	19	Discuss limitations of the study, taking into account sources of potential bias or imprecision. Discuss both direction and magnitude of any potential bias<br>**Location in manuscript: Discussion in Line 301-313**
Interpretation	20	Give a cautious overall interpretation of results considering objectives, limitations, multiplicity of analyses, results from similar studies, and other relevant evidence<br>**Location in manuscript: Discussion in Line 242-250**
Generalisability	21	Discuss the generalisability (external validity) of the study results<br>**Location in manuscript: Discussion in Line 242-250, 276-283**
**Other information**		
Funding	22	Give the source of funding and the role of the funders for the present study and, if applicable, for the original study on which the present article is based<br>**Location in manuscript: Funding declaration**

*Give information separately for cases and controls.

Note: An Explanation and Elaboration article discusses each checklist item and gives methodological background and published examples of transparent reporting. The STROBE checklist is best used in conjunction with this article (freely available on the Web sites of PLoS Medicine at http://www.plosmedicine.org/, Annals of Internal Medicine at http://www.annals.org/, and Epidemiology at http://www.epidem.com/). Information on the STROBE Initiative is available at http://www.strobe-statement.org.
