# Supplementary material for: Comparison of serum vitamin D level and vitamin D receptor gene FokI polymorphism in leprosy patients with and without trophic ulcers: A case-control study
Source: PLoS Negl Trop Dis. 2026 Apr 10;20(4):e0014205. doi: 10.1371/journal.pntd.0014205 (PMC13086432; doi:10.1371/journal.pntd.0014205)
Supplement: S1 Data — (DOCX) [file pntd.0014205.s003.docx]

https://doi.org/10.6084/m9.figshare.30999766.v2
