# Supplementary material for: Comparison of serum vitamin D level and vitamin D receptor gene FokI polymorphism in leprosy patients with and without trophic ulcers: A case-control study
Source: PLoS Negl Trop Dis. 2026 Apr 10;20(4):e0014205. doi: 10.1371/journal.pntd.0014205 (PMC13086432; doi:10.1371/journal.pntd.0014205)
Supplement: Attachment — Submitted filename: PNTD-D-26-00033_reviewer.pdf [file pntd.0014205.s004.pdf]

PLOS Neglected Tropical Diseases

Comparison of serum vitamin D level and vitamin D receptor gene FokI polymorphism in leprosy patients with and without trophic ulcers: A case-control study

--Manuscript Draft--

**Manuscript Number:**	PNTD-D-26-00033
**Full Title:**	Comparison of serum vitamin D level and vitamin D receptor gene FokI polymorphism in leprosy patients with and without trophic ulcers: A case-control study
**Short Title:**	Vitamin D level and VDR FokI polymorphism in leprosy trophic ulcer
**Article Type:**	Research Article
**Keywords:**	trophic ulcer; leprosy; vitamin D; FokI polymorphism; vitamin D receptor gene polymorphisms; neglected tropical disease
**Abstract:**	Background<br>Alterations in the vitamin D axis have been implicated in chronic wounds, but their role in leprosy-related trophic ulcers (TU) is unclear. This study compared serum vitamin D levels and VDRFokI (rs2228570) polymorphism between leprosy patients with and without TU, assessed their association with ulcer presence, and examined the correlation between vitamin D levels and ulcer severity.<br>Methods<br>This case-control study involved 82 adult leprosy patients (41 with TU, 41 without) treated at a tertiary referral hospital in Jakarta, Indonesia. Serum 25-hydroxyvitamin D [25(OH)D] levels were measured using chemiluminescence assay, and FokI polymorphism was analyzed using polymerase chain reaction-restriction fragment length polymorphism (PCR-RFLP). Ulcer severity was evaluated using the Pressure Ulcer Scale for Healing (PUSH). Associations were assessed through bivariate and multivariate analyses.<br>Results<br>Patients with TU had significantly lower mean serum 25(OH)D levels than those without TU (13.14 vs. 20.18 ng/mL, $p < 0.001$). The homozygous mutant FokI genotype (ff) was more frequent in the TU group ($p = 0.043$). In multivariable analysis, both low vitamin D status (adjusted odds ratio [aOR] 4.15; 95% confidence interval [CI] 1.41–12.24; $p = 0.010$) and the FokI polymorphism (aOR 7.86; 95% CI 1.80–34.36; $p = 0.006$) remained independently associated with TU. Vitamin D levels showed a weak inverse correlation with PUSH scores ($r = –0.312$, $p = 0.047$).<br>Conclusions<br>Lower serum vitamin D status and the FokI polymorphism were independently associated with TU in leprosy, and vitamin D levels were inversely correlated with ulcer severity. While genetic variation is non-modifiable, vitamin D status represents a potentially modifiable factor; hence, these findings suggest that assessment of of vitamin D status may be considered as part of comprehensive ulcer care in leprosy.
**Additional Information:**	
**Question**	**Response**
**Government Employee**<br><br>Are you or any of the contributing authors an employee of the United States government?<br><br>Manuscripts authored by one or more US Government employees are not copyrighted, but are licensed under a CC0 Public Domain Dedication, which allows unlimited distribution and reuse of the	No - No authors are employees of the U.S. government.

Powered by Editorial Manager® and ProduXion Manager® from Aries Systems Corporation

article for any lawful purpose. This is a legal requirement for US Government employees.<br><br>This will be typeset if the manuscript is accepted for publication.	
**Financial Disclosure**<br><br>Enter a financial disclosure statement that describes the sources of funding for the work included in this submission. Review the submission guidelines for detailed requirements. View published research articles from *PLOS Neglected Tropical Diseases* for specific examples.<br><br>This statement is required for submission and **will appear in the published article** if the submission is accepted. Please make sure it is accurate.<br><br>**Funded studies**<br>Enter a statement with the following details:<br>• Initials of the authors who received each award<br>• Grant numbers awarded to each author<br>• The full name of each funder<br>• URL of each funder website<br>• Did the sponsors or funders play any role in the study design, data collection and analysis, decision to publish, or preparation of the manuscript?<br><br>Did you receive funding for this work?	Yes
Please add funding details.<br>as follow-up to "**Financial Disclosure**<br><br>Enter a financial disclosure statement that describes the sources of funding for the work included in this submission. Review the submission guidelines for detailed requirements. View published research articles from *PLOS Neglected Tropical Diseases* for specific examples.<br><br>This statement is required for submission	The study is funded by the Directorate of Research and Development, Universitas Indonesia under the Hibah PUTI 2023 scheme (NKB-689/UN2.RST/HKP.05.00/2023). The funders had no role in study design, data collection and analysis, decision to publish, or preparation of the manuscript.

and **will appear in the published article** if the submission is accepted. Please make sure it is accurate.<br><br>**Funded studies**<br>Enter a statement with the following details:<br>• Initials of the authors who received each award<br>• Grant numbers awarded to each author<br>• The full name of each funder<br>• URL of each funder website<br>• Did the sponsors or funders play any role in the study design, data collection and analysis, decision to publish, or preparation of the manuscript?<br><br>Did you receive funding for this work?"	
Please select the country of your main research funder (please select carefully as in some cases this is used in fee calculation).<br>as follow-up to "**Financial Disclosure**<br><br>Enter a financial disclosure statement that describes the sources of funding for the work included in this submission. Review the submission guidelines for detailed requirements. View published research articles from *PLOS Neglected Tropical Diseases* for specific examples.<br><br>This statement is required for submission and **will appear in the published article** if the submission is accepted. Please make sure it is accurate.<br><br>**Funded studies**<br>Enter a statement with the following details:<br>• Initials of the authors who received each award<br>• Grant numbers awarded to each author<br>• The full name of each funder<br>• URL of each funder website<br>• Did the sponsors or funders play any role in the study design, data collection and analysis, decision to publish, or preparation of the manuscript?	INDONESIA - ID

Powered by Editorial Manager® and ProduXion Manager® from Aries Systems Corporation

Did you receive funding for this work?"	
**Competing Interests**<br><br>On behalf of all authors, disclose any competing interests that could be perceived to bias this work.<br><br>This statement will be typeset if the manuscript is accepted for publication.<br><br>Review the instructions link below and PLOS NTDs' competing interests policy to determine what information must be disclosed at submission.	The authors have no competing interests to declare.
**Data Availability**<br><br>Provide a **Data Availability Statement** in the box below. This statement should detail where the data used in this submission can be accessed. This statement will be typeset if the manuscript is accepted for publication.<br><br>Before publication, authors are required to make all data underlying their findings fully available, without restriction. Review our PLOS Data Policy page for detailed information on this policy. Instructions for writing your Data Availability statement can be accessed via the Instructions link below.	All relevant data are within the manuscript and its Supporting Information files.

Powered by Editorial Manager® and ProduXion Manager® from Aries Systems Corporation

Manuscript

Click here to access/download;Manuscript;Manuscript Vit D Leprosy Ulcer - PLOS Negl Trop Dis_05012026.pdf

Comparison of serum vitamin D level and vitamin D receptor gene *FokI* polymorphism in leprosy patients with and without trophic ulcers: A case-control study

Short title: Vitamin D level and *VDR FokI* polymorphism in leprosy trophic ulcer

Paulus Anthony Halim[1]; Sondang P. Sirait[1*]; Eliza Miranda[1]; Yulia Ariani[2,3]; Wresti Indriatmi[1]; Hok Bing Thio[4]

[1]Department of Dermatology and Venereology, Faculty of Medicine Universitas Indonesia, Dr. Cipto Mangunkusumo Hospital, Jakarta, Indonesia

[2]Department of Medical Biology, Faculty of Medicine, Universitas Indonesia, Jakarta, Indonesia

[3]Human Genetic Research Center, Indonesian Medical Education and Research Institute (IMERI), Universitas Indonesia, Jakarta, Indonesia

[4]Department of Dermatology, Erasmus University Medical Center, Rotterdam, The Netherlands

*Corresponding author:

E-mail: sondang_dr@yahoo.com (SPS)

SPS and EM are Joint Senior Authors.

Abstract

Background: Alterations in the vitamin D axis have been implicated in chronic wounds, but their role in leprosy-related trophic ulcers (TU) is unclear. This study compared serum vitamin D levels and *VDR FokI* (rs2228570) polymorphism between leprosy patients with and without TU, assessed their association with ulcer presence, and examined the correlation between vitamin D levels and ulcer severity.

Methods: This case-control study involved 82 adult leprosy patients (41 with TU, 41 without) treated at a tertiary referral hospital in Jakarta, Indonesia. Serum 25-hydroxyvitamin D [25(OH)D] levels were measured using chemiluminescence assay, and *FokI* polymorphism was analyzed using polymerase chain reaction-restriction fragment length polymorphism (PCR-RFLP). Ulcer severity was evaluated using the Pressure Ulcer Scale for Healing (PUSH). Associations were assessed through bivariate and multivariate analyses.

Results: Patients with TU had significantly lower mean serum 25(OH)D levels than those without TU (13.14 vs. 20.18 ng/mL, $p < 0.001$). The homozygous mutant *FokI* genotype (*ff*) was more frequent in the TU group ($p = 0.043$). In multivariable analysis, both low vitamin D status (adjusted odds ratio [aOR] 4.15; 95% confidence interval [CI] 1.41–12.24; $p = 0.010$) and the *FokI* polymorphism (aOR 7.86; 95% CI 1.80–34.36; $p = 0.006$) remained independently associated with TU. Vitamin D levels showed a weak inverse correlation with PUSH scores ($r = –0.312$, $p = 0.047$).

Conclusions: Lower serum vitamin D status and the *FokI* polymorphism were independently associated with TU in leprosy, and vitamin D levels were inversely correlated with ulcer severity. While genetic variation is non-modifiable, vitamin D status represents a potentially modifiable factor; hence, these findings suggest that assessment of of vitamin D status may be considered as part of comprehensive ulcer care in leprosy.

Author Summary

Leprosy can result in chronic wounds (trophic ulcers) that are difficult to heal. In this study, leprosy patients with ulcers had lower vitamin D levels than those without ulcers, and lower levels were linked to greater ulcer severity. A variant of the vitamin D receptor gene (*FokI* polymorphism), which produces a receptor with reduced transcriptional activity, was also more frequent among patients with ulcers, suggesting a genetic contribution to ulcer susceptibility. While genetic factors are non-modifiable, vitamin D deficiency is potentially correctable. Assessment of vitamin D status may therefore be relevant as part of comprehensive ulcer care in people affected by leprosy.

Introduction

Leprosy, or Hansen's disease (HD), is a chronic infection caused by *Mycobacterium leprae* and *M. lepromatosis*, that mainly affects the skin and peripheral nerves. Although global control efforts have reduced incidence, the disease remains endemic in countries like Brazil, India, and Indonesia, that together accounts for approximately 80% of new cases worldwide [1]. Vulnerable populations, particularly refugee and immigrant groups, also carry a disproportionately high leprosy burden. A recent work reported that 25.9% of all identified dermatological conditions in these populations were attributable to leprosy [2]. In 2023, Indonesia reported 14,376 new cases, with 3.0 new grade 2 disability (G2D) cases per million population, far exceeding the World Health Organization (WHO) 2030 target of 0.12 [1]. One of the most common cause of disability among leprosy patients is trophic ulcer (TU), affecting 10–20% of patients [3,4]. These chronic wounds, often on the plantar surface, result from neuropathy, foot deformities, impaired sensation, and

comorbidities [5]. They are notoriously difficult to manage and are associated with frequent recurrence, infection, and even malignant transformation [6].

Vitamin D, a secosteroid hormone, has demonstrated a key role in immune modulation and wound healing [7,8]. In damaged tissue, the circulating form, 25-hydroxyvitamin D [25(OH)D], is converted to calcitriol, binds to the vitamin D receptor (VDR), and induces antimicrobial peptides and cytokines involved in tissue repair [9–11]. Vitamin D deficiency has been linked to poor healing in chronic wounds (e.g., diabetic and venous ulcers) [12–14], with some studies showing improvement following supplementation [15,16].

Various genetic polymorphisms of the *VDR* gene may influence receptor function and downstream effects of vitamin D. Among these, the *FokI* polymorphism (rs2228570) is unique in that it alters the translation initiation site, resulting in structural variation of the receptor [17,18]. The *ff* genotype produces a longer, less active receptor, and has been associated with leprosy susceptibility and chronic ulcers in other conditions [19–21].

While previous studies have linked vitamin D deficiency and *VDR* polymorphisms to diabetic foot ulcers [20,22] and leprosy risk [23–26], their role in leprosy-related TU remains unexplored. This study aims to assess differences in serum 25(OH)D levels and the distribution of the *FokI* polymorphism between leprosy patients with and without TU, assess their association with TU incidence, and determine the correlation between vitamin D levels and ulcer severity.

Materials and Methods

Ethics statement

The study protocol adhered to the Declaration of Helsinki and was approved by the Health Research Ethics Committee of the Faculty of Medicine, Universitas Indonesia – Dr. Cipto

Mangunkusumo Hospital (No. KET-82/UN2.F1/ETIK/PPM.00.02/2024). All participants provided written informed consent following an explanation of the study objectives, procedures, and risks.

Study design

The full study protocol has been previously published [27]. In brief, this single-center, observational, analytic case-control study was conducted at the Dermatology and Venereology Clinic of Dr. Cipto Mangunkusumo Hospital, Jakarta, Indonesia. Adult leprosy patients, with and without TU, were consecutively recruited.

Participants

Eligible participants were adults aged 18 years or older who met the World Health Organization (WHO) diagnostic criteria for leprosy, including newly diagnosed individuals, those currently receiving treatment, or those released from treatment. The case group included patients with TU meeting all of the following criteria: ulcer duration of at least four weeks without a history of recent sharp debridement or callus thinning; ulcer depth not exceeding the subcutaneous layer; and absence of secondary infection or only mild signs of infection (i.e., surrounding erythema <2 cm, minimal edema or purulent discharge, and no warmth on palpation). Exclusion criteria were: recent consumption of vitamin D supplements within the past four weeks; current signs or symptoms of leprosy reactions; or comorbidities that may confound ulcer healing or vitamin D metabolism, including systemic infections, diabetes mellitus, severe hepatic dysfunction, end-stage renal disease, malignancy, or autoimmune disorders.

Data taking

Structured interviews and physical examinations were conducted using standardized forms. Collected data included sociodemographic details, leprosy and ulcer history, duration of sun exposure, and smoking status. Physical examination included anthropometric measurements, ulcer characteristics, presence of deformities, and sensory testing using 0.2 g (blue) and 2 g (purple) Semmes-Weinstein monofilaments. Ulcer photographs were taken using an iPhone 12 camera under standardized conditions. Ulcer area was calculated by multiplying two perpendicular diameters measured with a digital caliper, with the largest ulcer per subject selected for analysis. Ulcer depth and severity were assessed using the Pressure Ulcer Scoring System (PUSS) and the Pressure Ulcer Scale for Healing (PUSH) tool, respectively [28].

Sample collection and laboratory procedures

Venous blood (6 mL) was collected in ethylenediaminetetraacetic acid (EDTA) and plain tubes, stored at 2–8°C, and processed within 2 hours. Serum 25(OH)D concentrations were determined from plain tube samples using a chemiluminescent immunoassay (Architect 25(OH)D kit; Abbott Diagnostics, IL, USA). The classification of vitamin D status was based on Endocrine Society guidelines (<20 ng/mL for deficiency, 20–30 ng/mL for insufficiency, >30 ng/mL for sufficiency) [29], as locally validated cutoffs are unavailable. Severe deficiency was defined as <10 ng/mL in line with recent evidence [30].

The full procedure for *FokI* genotyping using the PCR–restriction fragment length polymorphism (PCR-RFLP) method is detailed in the published protocol [27]. In short, genomic DNA was extracted from EDTA blood using the salting-out method, followed by PCR with primers described by Soroush et al. [19]. The amplified products were digested with the *FokI*

enzyme (New England Biolabs, MA, USA) and visualized on 2% agarose gel. Genotypes were determined based on band patterns (S1 Fig): 192 and 58 bp for the homozygous mutant genotype (*ff*), 250, 192, and 58 bp for the heterozygous genotype (*Ff*), and a single 250 bp fragment for the homozygous wild-type genotype (*FF*).

Sample size estimates

Sample size was calculated to detect differences in serum vitamin D levels, the frequency of *FokI* polymorphism, and correlations with ulcer severity, using standard formulas with 80% power and 5% significance. Based on prior data, a minimum of 41 subjects per group was required, yielding a total of 82 participants [27].

Statistical analysis

Data were analyzed using STATA v16.0 (StataCorp LLC, College Station, TX, USA) and SNPStats (Catalan Institute of Oncology, Barcelona, Spain). Bivariate and multivariate analyses were conducted and significance was considered at $p < 0.05$. Variables with $p < 0.25$ in bivariate analysis were deemed eligible for inclusion in the multivariate logistic regression model, following a purposeful selection approach. All selected variables were entered into the final model to allow adjustment for potential confounding.

Results

Sociodemographic and clinical characteristics

A total of 82 participants were recruited between March and July 2024, during the Indonesian dry season, comprising 41 leprosy patients with TU and 41 without. The selection

process is summarized in the study flow diagram (Fig 1). Sociodemographic characteristics are presented in Table 1. No statistically significant differences were observed between the groups in terms of sex, age, occupation, marital status, smoking history, or duration of sun exposure. However, patients with TU had significantly lower education levels ($p < 0.001$) and a higher proportion of household incomes below the regional minimum wage ($p = 0.010$).

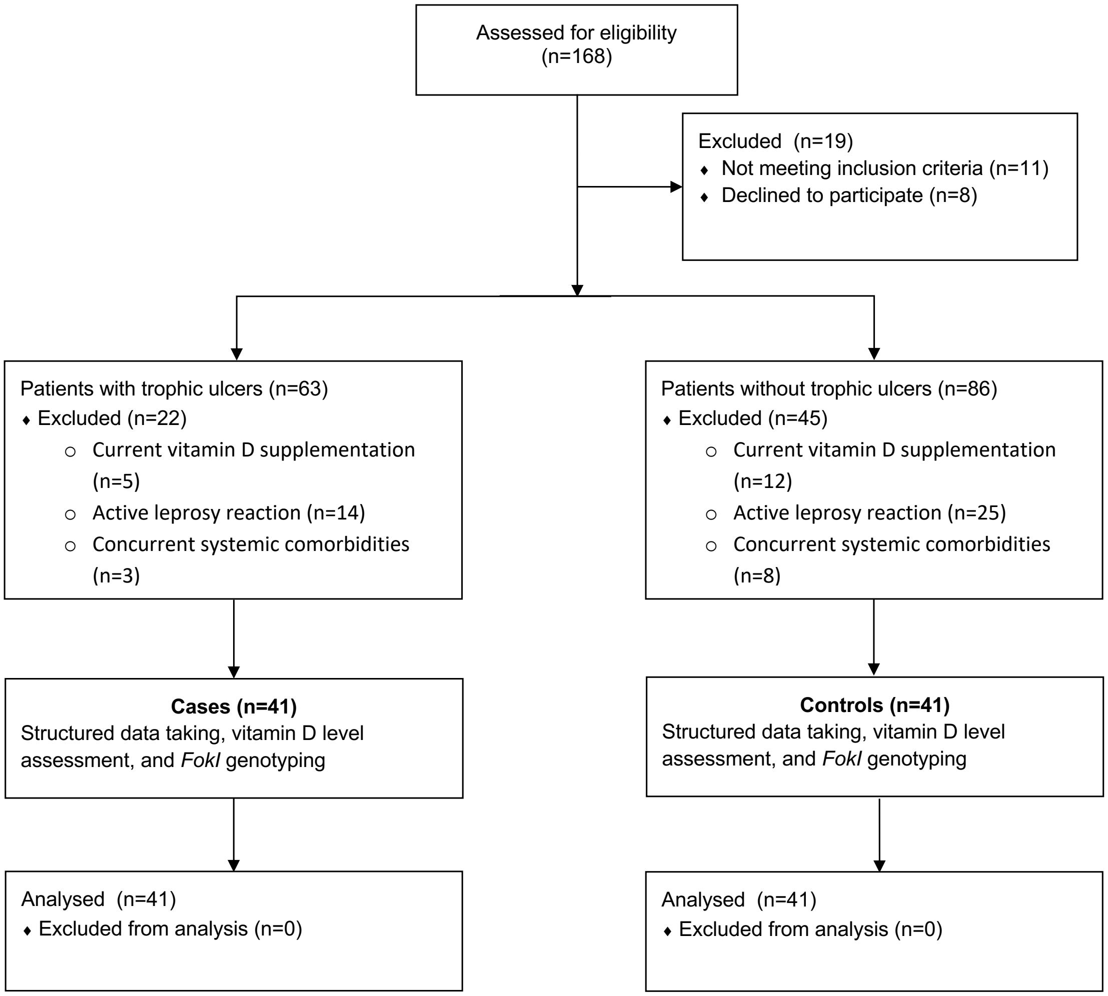

Fig 1. Study flow diagram.

Table 1. Sociodemographic characteristics of study subjects.

Characteristics	Leprosy with TU (n=41)	Leprosy without TU (n=41)	p-value
Sex, n (%)			0.822[a]
Male	24 (58.5)	25 (61.0)	
Female	17 (41.5)	16 (39.0)	
Age, years (mean±SD)	43.83 ± 11.38	40.83 ± 13.52	0.280[b]
Ethnic group, n (%)			0.218[a]
Javanese	15 (36.6)	14 (34.1)	
Betawi	10 (24.4)	9 (22.0)	
Sundanese	11 (26.8)	6 (14.6)	
Others	5 (12.2)	12 (29.3)	
Education level, n (%)			**<0.001[a]**
None or primary	18 (43.9)	3 (7.3)	
Secondary	20 (48.8)	23 (56.1)	
Tertiary	3 (7.3)	15 (36.6)	
Occupation, n (%)			0.334[a]
Unemployed	18 (43.9)	14 (34.1)	
Employee	18 (43.9)	17 (41.5)	
Self-employed	5 (12.2)	10 (24.4)	
Marital status, n (%)			0.414[a]
Unmarried	7 (17.1)	10 (24.4)	
Married	34 (82.9)	31 (75.6)	
Household income, n (%)			**0.010[a]**
< Jakarta RMW	33 (80.5)	22 (53.7)	
≥ Jakarta RMW	8 (19.5)	19 (46.3)	
Smoking history, n (%)			0.822[a]
No	24 (58.5)	25 (61.0)	
Yes	17 (41.5)	16 (39.0)	
Sun exposure duration, hours/week [median (range)]	5.0 (0.5–35.0)	5.5 (1.0–42.0)	0.613[c]

RMW, 2024 Jakarta monthly regional minimum wage (Rp 5,067,381 or circa US$ 301); SD, standard deviation; TU, trophic ulcer; [a]*Chi-square* test; [b]independent t-test; [c]Mann-Whitney U test

The clinical characteristics of both groups are detailed in Table 2. Subjects in the TU group had significantly longer disease duration (median 10 vs. 2.25 years, $p < 0.001$), higher prevalence of sensory neuropathy in the hands (97.6% vs. 51.2%, $p < 0.001$) and feet (100% vs. 78%, $p = 0.002$), and higher prevalence of non-ulcer deformities (70.7% vs. 12.2%, $p < 0.001$). No significant differences were observed in nutritional status, disease classification (WHO and Ridley-Jopling), treatment status, history of leprosy reactions, and hypertension

Table 2. Clinical characteristics of study subjects.

Characteristics	Leprosy with TU (n=41)	Leprosy without TU (n=41)	p-value
Body mass index, kg/m^2 (mean±SD)	21.94 ± 3.67	23.19 ± 3.94	0.140[a]
Nutritional status, n (%)			0.284[b]
Underweight	7 (17.1)	3 (7.3)	
Normal weight	20 (48.8)	17 (41.5)	
Overweight	3 (7.3)	7 (17.1)	
Obesity	11 (26.8)	14 (34.1)	
Duration of leprosy, n (%)			**<0.001[b]**
≤ 1 year	2 (4.9)	11 (26.8)	
1–5 year	14 (34.1)	22 (53.7)	
>5 year	15 (61.0)	8 (19.5)	
WHO classification, n (%)			1.000[c]
Paucibacillary	0 (0)	1 (2.4)	
Multibacillary	41 (100)	40 (97.6)	
Ridley-Jopling classification, n (%)			1.000[c]
BT	16 (39.0)	15 (36.6)	
BB	2 (4.9)	3 (7.3)	
BL	13 (31.7)	14 (34.1)	
LL	10 (24.4)	9 (22.0)	
Treatment status, n (%)			0.077[b]
Under treatment	16 (39.0)	24 (58.5)	
Released from treatment	25 (61.0)	17 (41.5)	
History of reaction, n (%)			0.201[c]
No	5 (12.2)	6 (14.6)	
Type 1	21 (51.2)	28 (68.3)	
Type 2	14 (34.2)	6 (14.6)	
Both	1 (2.4)	1 (2.4)	
Hand sensory neuropathy, n (%)			**<0.001[c]**
No	1 (2.4)	20 (48.8)	
Yes	40 (97.6)	21 (51.2)	

Foot sensory neuropathy, n (%)			**0.002[c]**
No	0 (0)	9 (22)	
Yes	41 (100)	32 (78)	
Non-ulcer deformity, n (%)			**<0.001[b]**
No	12 (29.3)	36 (87.8)	
Yes	29 (70.7)	5 (12.2)	
Hypertension, n (%)			0.135[b]
No	27 (65.9)	33 (80.5)	
Yes	14 (34.1)	8 (19.5)	

TU, trophic ulcer; SD, standard deviation; [a]Independent t-test; [b]Chi-square test; [c]Fisher's exact test

Ulcer characteristics

There were 69 ulcers among the 41 subjects with TU. For consistency in analysis, only the largest ulcer per patient was evaluated, with aggregated data presented in Table 3. The median ulcer area was 1.89 cm^2, and the majority (78.1%) extended to the subcutaneous tissue. Most ulcers were located on the lower extremities (90.2%), particularly on the forefoot (41.5%). The median ulcer duration was four months, and the median PUSH score was 7 (range 3–14). The majority of ulcers exhibited mild to moderate exudate (70.7%) and had a granulation tissue base (73.2%). Callus formation surrounding the ulcer was observed in 82.9% of cases.

Table 3. Characteristics of the largest ulcer among the trophic ulcer group (n=41)

Characteristics	Value
Vertical diameter, cm [median (range)]	1.43 (0.20–9.37)
Horizontal diameter, cm [median (range)]	1.42 (0.20–7.05)
Area, cm^2 [median (range)]	1.89 (0.04–41.23)
Depth, n (%)	
Grade 2 – dermis	9 (21.9)
Grade 3 – subcutaneous	32 (78.1)
Ulcer duration, n (%)	
1–6 months	25 (61.0)
6 months– 2 years	11 (26.8)
2–5 years	4 (9.8)
>5 years	1 (2.4)
Location, n (%)	
Forefoot	17 (41.5)
Ankle	5 (12.2)
Hallux	5 (12.2)
Midfoot	3 (7.3)
Toes	2 (4.9)
Hindfoot	2 (4.9)
Dorsum of foot	1 (2.4)
Palm	2 (4.9)
Fingers	2 (4.9)
Others	2 (4.9)
Total PUSH score [median (range)]	7 (3–14)
Exudate subscore, n (%)	
0 – None	11 (26.8)
1 – Mild	16 (39.0)
2 – Moderate	13 (31.7)
3 – Severe	1 (2.4)
Wound bed subscore, n (%)	
1 – Epithelial tissue	3 (7.3)
2 – Granulation tissue	30 (73.2)
3 – Slough	7 (17.1)
4 – Necrotic tissue	1 (2.4)
Peri-ulcer callus formation, n (%)	
No	7 (17.1)
Yes	34 (82.9)

Serum 25(OH)D levels

The mean serum 25(OH)D concentration was notably lower in individuals with trophic ulcers (TU) compared to those without (13.14 ± 5.88 ng/mL vs. 20.18 ± 7.19 ng/mL, $p < 0.001$). Furthermore, vitamin D deficiency or severe deficiency was significantly more prevalent among patients in the TU group (82.9% vs. 36.6%, $p < 0.001$), as shown in Table 4.

Table 4. Vitamin D status and *FokI* polymorphism genotype and allele frequencies in leprosy patients with and without trophic ulcers.

	**Leprosy with TU (n = 41)**	**Leprosy without TU (n = 41)**	**p-value**
Vitamin D status, n (%)			**<0.001[a]**
Sufficiency	0 (0.0)	1 (2.4)	
Insufficiency	7 (17.1)	25 (61.0)	
Deficiency	20 (48.8)	9 (22.0)	
Severe deficiency	14 (34.1)	6 (14.6)	
FokI genotype, n (%)			**0.043[b]**
FF	8 (19.5)	14 (34.2)	
Ff	22 (53.7)	24 (58.5)	
ff	11 (26.8)	3 (7.3)	
Allele, n (%)			**0.028[b]**
F	38 (46.3)	52 (63.4)	
f	44 (53.7)	30 (36.6)	

TU, trophic ulcer; [a]Fisher's exact test, [b]Chi-square test

FokI polymorphism

Genotype and allele frequencies of the *FokI* polymorphism are presented in Table 4. Genotype distributions in both the case ($p = 0.76$) and control ($p = 0.18$) groups conformed to Hardy–Weinberg equilibrium. The homozygous mutant genotype (*ff*) was nearly four times more prevalent in patients with TU than in those without (26.8% vs. 7.3%, $p = 0.043$). In contrast, the wild-type genotype (*FF*) was more frequently observed in the non-TU group (34.2% vs. 19.5%).

The mutant allele (*f*) was also more frequently observed in the TU group than in the control group (53.7% vs. 36.6%, $p = 0.028$).

Association between vitamin D and *FokI* polymorphism with TU

In bivariate analysis, vitamin D status, *FokI* polymorphism, educational level, household income, leprosy duration, and of non-ulcer deformity were associated with TU (Table 5). These variables, together with nutritional status, were included in the multivariable logistic regression to control for potential confounding. After adjustment, low vitamin D status remained independently associated with TU (adjusted odds ratio [aOR] 4.15; 95% CI: 1.41–12.24; p = 0.010), as did the presence of the *FokI* polymorphism (aOR 7.86; 95% CI: 1.80–34.36; p = 0.006). Educational level showed a protective association with TU, while non-ulcer deformity remained positively associated. Leprosy duration did not remain statistically significant after adjustment but was retained in the model due to its clinical relevance as a potential confounder.

Table 5. Multivariate analyses of variables associated with trophic ulcers.

**Variable**	**Bivariate analysis**		**Multivariate analysis**	
	Crude OR (95% CI)	p-value	Adjusted OR (95% CI)	p-value
Vitamin D status	3.26 (1.71–6.20)	**<0.001**	4.15 (1.41-12.24)	**0.010**
FokI polymorphism	2.32 (1.13–4.76)	**<0.001**	7.86 (1.80-34.36)	**0.006**
Education	0.19 (0.08–0.44)	**<0.001**	0.19 (0.04–0.92)	**0.039**
Household income	0.28 (0.10-0.75)	**0.012**	0.49 (0.06-3.93)	0.504
Nutritional status	0.73 (0.48–1.11)	0.137	1.05 (0.50–2.24)	0.893
Leprosy duration	1.15 (1.05-1.26)	**0.004**	1.13 (0.97-1.31)	0.119
Presence of non-ulcer deformity	17.40 (5.50-55.07)	**<0.001**	6.72 (1.10-41.18)	**0.039**

OR, odds ratio; 95% CI, 95% confidence interval

Correlation between vitamin D levels and ulcer severity

A weak but statistically significant inverse correlation was found between serum 25(OH)D levels and PUSH scores among leprosy patients with TU (Pearson's $r = –0.312$, $p = 0.047$), as shown in Fig 2.

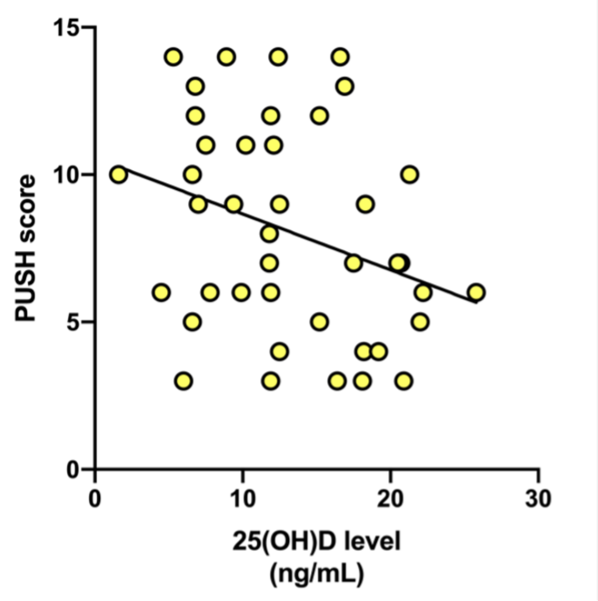

Fig 2. Correlation between serum 25(OH)D levels and trophic ulcer severity, as assessed using the Pressure Ulcer Scale for Healing (PUSH) scoring.

Genetic model analysis

Different inheritance models of the *FokI* polymorphism were evaluated for their association with TU (Table 6). Statistically significant associations were observed under the codominant ($p = 0.037$), recessive ($p = 0.016$), and log-additive ($p = 0.017$) models. The recessive model showed the best fit based on the lowest Akaike and Bayesian Information Criterion values. In this model, patients with the homozygous mutant genotype (*ff*) had a 4.64-fold higher risk of developing TU compared to those with *FF* or *Ff* genotypes.

Table 6. Genetic models of *FokI* polymorphism associated with trophic ulcer among leprosy patients.

Model	Genotype	Leprosy with TU (n = 41)	Leprosy without TU (n = 41)	Adjusted OR (IK 95%)	p-value	AIC	BIC
Codominant	*FF*	8 (19.5%)	14 (34.1%)	Reference	**0.037**	113.1	120.3
	Ff	22 (53.7%)	24 (58.5%)	1.60 (0.56–4.56)			
	ff	11 (26.8%)	3 (7.3%)	6.42 (1.37–30.06)			
Dominant	*FF*	8 (19.5%)	14 (34.1%)	Reference	0.130	115.4	120.2
	Ff – ff	33 (80.5%)	27 (65.8%)	2.14 (0.78–5.85)			
Recessive	*FF – Ff*	30 (73.2%)	38 (92.7%)	Reference	**0.016**	111.9	116.7
	ff	11 (26.8%)	3 (7.3%)	4.64 (1.19–18.16)			
Overdominant	*FF – ff*	19 (46.3%)	17 (41.5%)	Reference	0.660	117.5	122.3
	Ff	22 (53.7%)	24 (58.5%)	0.82 (0.34–1.96)			
Log-additive	–	–	–	2.32 (1.13–4.76)	**0.017**	112	116.8

TU, trophic ulcer; OR, odds ratio; AIC, Akaike Information Criterion; BIC, Bayesian Information Criterion.

Discussion

This study highlights an observed association between vitamin D status and receptor polymorphism with TU development among leprosy patients. Serum 25(OH)D levels were lower and the *FokI* polymorphism was more frequent in patients with TU, and both remained associated with TU after adjustment for confounders. A modest inverse correlation was also found between vitamin D levels and ulcer severity. However, causality cannot be inferred due to the case-control design.

Despite the absence of sample matching, comparable age and sex distributions between groups likely minimized confounding. Some sociodemographic disparities persisted, with significantly more patients in the TU group having lower educational attainment and household incomes below the regional minimum wage. These findings align with prior evidence that low socioeconomic status impairs access to healthcare and delays ulcer prevention and management [5]. Clinically, patients with TU had a significantly longer disease duration and higher prevalence

of sensory neuropathy and deformities. These features are well-established risk factors for ulcer formation due to repeated unnoticed trauma and altered biomechanical pressure points [3,5,31], particularly on weight-bearing areas like the forefoot, which was the most common ulcer site in this study. The median ulcer duration of four months, with a substantial portion persisting for several years, underscores the chronic and refractory nature of TU in leprosy.

Patients with TU exhibited significantly lower serum 25(OH)D levels, supporting our primary hypothesis. Vitamin D status also remained also associated with TU after adjustment for potential confounders. To our knowledge, this is the first investigation to explore this association specifically in individuals with leprosy. As this was a case-control study, temporal direction cannot be determined, and the findings should not be interpreted as evidence of causality. Low vitamin D levels have similarly been associated with impaired healing in other chronic ulcers, including diabetic, venous, and pressure ulcers, suggesting a broader role of vitamin D in wound biology [12–14,32,33]. Furthermore, in our study, lower vitamin D levels correlated with increased TU severity, consistent with findings in diabetic foot ulcers (DFU) [12,34,35]. This pattern suggests that reduced vitamin D may be linked to more severe chronic ulcers.

Vitamin D, through its active form that binds to the cytoplasmic vitamin D receptor, regulates genes involved in antimicrobial peptide production, inflammatory response, and tissue repair. Various vitamin D-dependent pathways are engaged throughout these processes, thus disturbances caused by low vitamin D levels or receptor polymorphisms can interfere with these functions. In leprosy, two Brazilian studies have shown that both vitamin D deficiency and *VDR* polymorphisms are associated with increased disease susceptibility [36,37]. Evidence from chronic ulcer research also indicates that vitamin D contributes to wound healing through effects on innate immunity, cytokine regulation, angiogenesis, and keratinocyte proliferation [7,9,10].

Vitamin D supplementation has been linked to improved outcomes in chronic ulcers [15,38], and agents acting downstream of vitamin D have shown clinical benefit. In a randomized trial, Miranda et al. [39] reported that topical LL-37, a peptide induced by vitamin D signaling, accelerated wound granulation in DFU.

Furthermore, vitamin D influences several pathways that may contribute to TU formation in leprosy. Reduced vitamin D activity weakens vitamin D-dependent innate immune pathways, lowering LL-37 and other antimicrobial peptides [11,40]. This impaired response may compromise cutaneous host defence and contribute to the microbiome dysbiosis and metabolic disturbances reported in TU [41]. Adequate vitamin D signalling also supports TLR2/1-mediated macrophage killing of *M. leprae* [42] and promotes nerve repair through Schwann cells [43]; therefore, deficiency may exacerbate neural damage. As neuropathy progresses, sensory loss permits repeated unrecognised trauma, while autonomic fiber involvement can reduce local perfusion and sweating, weakening the epidermal barrier [44]. These fibers also release neuropeptides that promote vasodilation, chemotaxis, and keratinocyte repair, and loss of these signals has been shown to delay wound healing in diabetic neuropathy [45]. While a similar neuropeptide-related disturbance is plausible in leprosy, it remains understudied.

Although seasonal variation in vitamin D levels is well recognized in higher-latitude countries, its impact in this study is likely minimal. Indonesia, an equatorial country, receives relatively constant daylight duration and high UV indices throughout the year, including in Jakarta where seasonal fluctuations are modest [46]. Studies from Indonesia and other Southeast Asian countries have not demonstrated meaningful seasonal differences in serum 25(OH)D levels [47,48]. In this study, all participants were recruited during the dry season, reducing intra-study

variation in UV exposure. While the wet season may theoretically reduce sun exposure, these factors indicate that seasonal bias is unlikely to have significantly affected our findings.

The homozygous mutant (*ff*) genotype and allele (*f*) of the *FokI* polymorphism more frequent among TU patients. Although the *FokI* polymorphism remained associated with TU after multivariable adjustment, the association appeared less robust than that of vitamin D status. suggesting that its clinical relevance may be modulated by modifiable factors such as vitamin D levels. Similar patterns have been reported in DFU, where associations observed in unadjusted analyses were attenuated after accounting for vitamin D status and other covariates [20].

Mechanistically, the *ff* genotype encodes a longer VDR isoform with reduced transcriptional activity [49]. Under the recessive model, individuals with the *Ff* genotype still produce the shorter, more active isoform, which may explain the limited biological effect of *FokI* heterozygosity. Reduced VDR activity in *ff* homozygotes could diminish vitamin D-dependent antimicrobial, immunomodulatory, and tissue repair pathways [7,9], contributing to neural damage or delayed wound healing in leprosy. Still, these effects may be insufficient to influence ulcer development without concurrent vitamin D deficiency. While the recessive model best fit our data, the log-additive was also significant, indicating a possible incremental effect of each mutant allele and hinting at a broader polygenic mechanism on ulcer susceptibility. Future genome-wide association studies (GWAS) are warranted to identify additional genetic loci involved in immune regulation, nerve damage, and wound repair as potential risk factors for TU in leprosy patients.

Strengths and limitations

This study is the first to investigate both serum vitamin D levels and *FokI* polymorphism in leprosy patients with and without TU. It also represents an early effort to explore the potential involvement of the vitamin D axis in TU development within this population. Nonetheless, several

limitations should be acknowledged. The single-center design may limit generalizability, and the case-control methodology precludes determination of temporal or causal relationships. Although recalled weekly sun exposure duration was similar between groups, reverse causality cannot be excluded, as chronic ulcers may restrict outdoor mobility and reduce effective sun exposure over time, potentially resulting in lower vitamin D levels. Several baseline characteristics differed between groups; however, clinically relevant variables were addressed through multivariable adjustment, although the modest sample size may have limited the precision of these estimates, particularly for genetic associations. Finally, only one *VDR* polymorphism was examined, while other potentially relevant *VDR* and non-*VDR* variants were not assessed and warrant future study.

Conclusion

This study demonstrates that lower serum vitamin D status and the *FokI* polymorphism were independently associated with the presence of TU in patients with leprosy, and that vitamin D levels were inversely correlated with ulcer severity. While genetic variation may contribute to ulcer susceptibility, vitamin D status represents a potentially modifiable parameter in clinical practice. These findings support consideration of vitamin D status assessment within comprehensive ulcer care in leprosy. However, prospective cohort studies and interventional trials are required to clarify temporal relationships and to determine whether correction of vitamin D deficiency can reduce ulcer incidence or severity in this population.

Acknowledgements

None.

References

1. World Health Organization. Global leprosy (Hansen disease) update, 2023: Elimination of leprosy disease is possible – Time to act! Weekly Epidemiological Record. 2024;99: 501–521. Available: https://www.who.int/publications/i/item/who-wer9937-501-521
2. Burke OM, Yang S, Beer J, Htet KZ, Elman SA. Infectious Dermatological Conditions Among Refugee and Immigrant Populations: A Systematic Review. Int J Dermatol. 2025;0: 1–16. doi:10.1111/ijd.70042
3. Upputuri B, Srikantam A, Mamidi RS. Comorbidities associated with non-healing of plantar ulcers in leprosy patients. PLoS Negl Trop Dis. 2020;14: e0008393. doi:10.1371/journal.pntd.0008393
4. de Almeida Gonçalves Sachett J, da Silva Moreira RM, Porto MCA, Nascimento TP, Monteiro WM. The marks of leprosy: disabilities caused by plantar lesions in discharged patients. Arch Dermatol Res. 2024;316. doi:10.1007/s00403-024-03227-2
5. Govindasamy K, Darlong J, Watson SI, Gill P. Prevalence of plantar ulcer and its risk factors in leprosy: a systematic review and meta-analysis. J Foot Ankle Res. 2023;16: 77. doi:10.1186/s13047-023-00674-4
6. Karthikeyan K, Thappa DM. Squamous cell carcinoma in plantar ulcers in leprosy: a study of 11 cases. Indian J Leprosy. 2003;75: 219–224.
7. Bikle DD. Role of vitamin D and calcium signaling in epidermal wound healing. J Endocrinol Invest. 2022;46: 205–212. doi:10.1007/s40618-022-01893-5
8. Schwalfenberg GK. A review of the critical role of vitamin D in the functioning of the immune system and the clinical implications of vitamin D deficiency. Mol Nutr Food Res. 2011;55: 96–108. doi:10.1002/MNFR.201000174

9. Oda Y, Tu CL, Menendez A, Nguyen T, Bikle DD. Vitamin D and calcium regulation of epidermal wound healing. J Steroid Biochem Mol Biol. 2016;164: 379–385. doi:10.1016/J.JSBMB.2015.08.011

10. Ding J, Kwan P, Ma Z, Iwashina T, Wang J, Shankowsky HA, et al. Synergistic effect of vitamin D and low concentration of transforming growth factor beta 1, a potential role in dermal wound healing. Burns. 2016;42: 1277–1286. doi:10.1016/J.BURNS.2016.03.009

11. Segaert S. Vitamin D regulation of cathelicidin in the skin: Toward a renaissance of vitamin D in dermatology? J Invest Dermatol. 2008;128: 773–775. doi:10.1038/jid.2008.35

12. Tang Y, Huang Y, Luo L, Xu M, Deng D, Fang Z, et al. Level of 25-hydroxyvitamin D and vitamin D receptor in diabetic foot ulcer and factor associated with diabetic foot ulcers. Diabetol Metab Syndr. 2023;15: 1–17. doi:10.1186/s13098-023-01002-3

13. Priyanto MH, Legiawati L, Saldi SRF, Yunir E, Miranda E. Comparison of vitamin D levels in diabetes mellitus patients with and without diabetic foot ulcers: An analytical observational study in Jakarta, Indonesia. Int Wound J. 2023;20: 2028–36. doi:10.1111/iwj.14066

14. Burkievcz CJC, Skare TL, Malafaia O, Nassif PAN, Ribas CSG, Santos LRP. Vitamin D deficiency in patients with chronic venous ulcers. Rev Col Bras Cir. 2012;39: 60–3. doi:22481708

15. Razzaghi R, Pourbagheri H, Momen-Heravi M, Bahmani F, Shadi J, Soleimani Z, et al. The effects of vitamin D supplementation on wound healing and metabolic status in patients with diabetic foot ulcer: A randomized, double-blind, placebo-controlled trial. J Diabetes Complications. 2017;31: 766–772. doi:10.1016/j.jdiacomp.2016.06.017

16. Wu X, Zeng J, Ye X, Peng M, Lan Y, Zhang S, et al. Effects of vitamin D supplementation on diabetic foot ulcer healing: a meta-analysis. Postgrad Med J. 2024; qgae107. doi:10.1093/postmj/qgae107

17. Uitterlinden AG, Fang Y, van Meurs JBJ, Pols HAP, van Leeuwen JPTM. Genetics and biology of vitamin D receptor polymorphisms. Gene. 2004;338: 143–156. doi:https://doi.org/10.1016/j.gene.2004.05.014

18. Saccone D, Asani F, Bornman L. Regulation of the vitamin D receptor gene by environment, genetics and epigenetics. Gene. 2015;561: 171–180. doi:10.1016/j.gene.2015.02.024

19. Usategui-Martín R, De Luis-Román DA, Fernández-Gómez JM, Ruiz-Mambrilla M, Pérez-Castrillón JL. Vitamin D Receptor (VDR) gene polymorphisms modify the response to vitamin D supplementation: A systematic review and meta-analysis. Nutrients. 2022;14. doi:10.3390/nu14020360

20. Soroush N, Radfar M, Hamidi AK, Abdollahi M, Qorbani M, Razi F, et al. Vitamin D receptor gene FokI variant in diabetic foot ulcer and its relation with oxidative stress. Gene. 2017;599: 87–91. doi:https://doi.org/10.1016/j.gene.2016.11.012

21. Lubis RD, Roesyanto-Mahadi ID, Siregar Y, Eyanoer PC. FokI Vitamin D receptor gene polymorphism in leprosy household contacts. Malay J Med Health Sci. 2021;17: 113–116.

22. Tang W, Chen D, Chen L, Liu G, Sun S, Wang C, et al. The correlation between serum vitamin D status and the occurrence of diabetic foot ulcers: a comprehensive systematic review and meta-analysis. Sci Rep. 2024;14: 21932. doi:10.1038/s41598-024-73133-0

23. Singh I, Lavania M, Pathak VK, Ahuja M, Turankar RP, Singh V, et al. VDR polymorphism, gene expression and vitamin D levels in leprosy patients from North Indian population. PLoS Negl Trop Dis. 2018;12: e0006823. doi:10.1371/journal.pntd.0006823

24. Pepineli AC, Alves HV, Tiyo BT, Macedo LC, Visentainer L, de Lima Neto QA, et al. Vitamin D receptor gene polymorphisms are associated with leprosy in Southern Brazil. Front Immunol. 2019;10: 1–6. doi:10.3389/fimmu.2019.02157

25. Darus NIM, Lubis RD, Jusuf NK. Analysis of serum vitamin D level in leprosy patients. Bali Med J. 2019;8: 795–799. doi:10.15562/bmj.v8i3.1453

26. Neela VSK, Suryadevara NC, Shinde VG, Pydi SS, Jain S, Jonnalagada S, et al. Association of Taq I, Fok I and Apa I polymorphisms in Vitamin D Receptor (VDR) gene with leprosy. Hum Immunol. 2015;76: 402–405. doi:10.1016/j.humimm.2015.04.002

27. Halim PA, Sirait SP, Miranda E, Ariani Y, Indriatmi W, Thio HB. Comparison of serum vitamin D level and vitamin D receptor gene FokI polymorphism in leprosy patients with and without trophic ulcers: protocol for a case-control study. F1000Res. 2025;14: 57. doi:10.12688/f1000research.157869.3

28. Gardner SE, Frantz RA, Bergquist S, Shin CD. A prospective study of the pressure ulcer scale for healing (PUSH). J Gerontol. 2005;60: 93–97. doi:10.1093/gerona/60.1.93

29. Holick MF, Binkley NC, Bischoff-Ferrari HA, Gordon CM, Hanley DA, Heaney RP, et al. Evaluation, treatment, and prevention of vitamin D deficiency: An endocrine society clinical practice guideline. Journal of Clinical Endocrinology and Metabolism. 2011;96: 1911–1930. doi:10.1210/jc.2011-0385

30. Giustina A, Bilezikian JP, Adler RA, Banfi G, Bikle DD, Binkley NC, et al. Consensus Statement on Vitamin D Status Assessment and Supplementation: Whys, Whens, and Hows. Endocr Rev. 2024;45: 625–654. doi:10.1210/endrev/bnae009

31. Riyaz N, Sehgal VN. Leprosy: trophic skin ulcers. Skinmed. 2017;15: 45–51.

32. Dai J, Yu M, Chen H, Chai Y. Association between serum 25-OH-vitamin D and diabetic foot ulcer in patients with type 2 diabetes. Front Nutr. 2020;7. doi:10.3389/fnut.2020.00109

33. Takahashi P, Kalava, Cha. Association between vitamin D and pressure ulcers in older ambulatory adults: results of a matched case-control study. Clin Interv Aging. 2011; 213. doi:10.2147/cia.s23109

34. Zubair M, Malik A, Meerza D, Ahmad J. 25-hydroxyvitamin D [25(OH)D] levels and diabetic foot ulcer: Is there any relationship? Diabetes Metab Syndr. 2013;7: 148–153. doi:10.1016/j.dsx.2013.06.008

35. Wang F, Zhou L, Zhu D, Yang C. A retrospective analysis of the relationship between 25-OH-vitamin D and diabetic foot ulcer. Diabetes Metab Syndr Obes. 2022;15: 1347–1355. doi:10.2147/DMSO.S358170

36. Paz JLP, Silvestre MDPSCA, Moura LS, Furlaneto IP, Rodrigues YC, Batista Lima KV, et al. Association of the polymorphism of the vitamin D receptor gene (VDR) with the risk of leprosy in the Brazilian Amazon. Biosci Rep. 2021;41: 1–9. doi:10.1042/BSR20204102

37. Dennison CL, de Oliveira LB, Fraga LA de O, e Lima RS, Ferreira JA, Clennon JA, et al. Mycobacterium leprae–helminth co-infections and vitamin D deficiency as potential risk factors for leprosy: A case–control study in south-eastern Brazil. Int J Infect Dis. 2021;105: 261–266. doi:10.1016/j.ijid.2021.02.048

38. Halschou-Jensen PM, Sauer J, Bouchelouche P, Fabrin J, Brorson S, Ohrt-Nissen S. Improved healing of diabetic foot ulcers after high-dose vitamin D: A randomized double-blinded clinical trial. Int J Low Extrem Wounds. 2023;22: 466–474. doi:10.1177/15347346211020268

39. Miranda E, Bramono K, Yunir E, Reksodiputro MH, Suwarsa O, Rengganis I, et al. Efficacy of LL-37 cream in enhancing healing of diabetic foot ulcer: a randomized double-blind controlled trial. Arch Dermatol Res. 2023;315: 2623–2633. doi:10.1007/s00403-023-02657-8

40. de Oliveira ALG, Chaves AT, Santos Cardoso M, Gomide Pinheiro GR, Parreiras de Jesus AC, de Faria Grossi MA, et al. Hypovitaminosis D and reduced cathelicidin are strongly correlated during the multidrug therapy against leprosy. Microb Pathog. 2020;147: 104373. doi:10.1016/j.micpath.2020.104373

41. Wang J, Wang B, Liang C, Jin C, Shen H. Analysis of lower-limb ulcers in participants with leprosy sequelae using metabolomics and 16S ribosomal DNA sequencing. Clin Cosmet Investig Dermatol. 2023;16: 3465–3480. doi:10.2147/CCID.S441000

42. Fonseca AB de L, Simon M do V, Cazzaniga RA, de Moura TR, de Almeida RP, Duthie MS, et al. The influence of innate and adaptative immune responses on the differential clinical outcomes of leprosy. Infect Dis Poverty. 2017;6: 1–8. doi:10.1186/s40249-016-0229-3

43. Alhamdi AA, Mackie S, Trueman RP, Rayner MLD. Pharmacologically targeting Schwann cells to improve regeneration following nerve damage. Front Cell Dev Biol. 2025;13: 1–13. doi:10.3389/fcell.2025.1603752

44. Khadilkar S V., Patil SB, Shetty VP. Neuropathies of leprosy. J Neurol Sci. 2021;420: 117288. doi:10.1016/j.jns.2020.117288

45. Theocharidis G, Veves A. Autonomic nerve dysfunction and impaired diabetic wound healing: The role of neuropeptides. Auton Neurosci. 2020;223: 102610. doi:10.1016/j.autneu.2019.102610

46. Badan Metereologi Klimatologi dan Geofisika. Informasi Iklim Perkotaan Jakarta dan Sekitarnya. 2025 [cited 27 Nov 2025]. Available: https://iklim.bmkg.go.id/id/urban-jakarta/

47. Koon Poh B, Rojroongwasinkul N, Khanh Le Nguyen B, Sandjaja S, Ruzita AT, Yamborisut U, et al. 25-Hydroxy-Vitamin D Demography and the Risk of Vitamin D insufficiency in the South East Asian Nutrition Surveys (SEANUTS). Asia Pac J Clin Nutr. 2016;25: 538–548. doi:10.6133/apjcn.092015.02

48. Aris N, Mitra AK, Wan Mohamed WMI, Wan Muda WAM, Jan Mohamed HJ. Effects of occupational sunlight exposure and monsoon season on vitamin D concentration among outdoor and indoor workers in Malaysia. Malays J Nutr. 2020;26: 425–439. doi:10.31246/mjn-2020-0038

49. Uitterlinden AG, Fang Y, van Meurs JBJ, Pols HAP, van Leeuwen JPTM. Genetics and biology of vitamin D receptor polymorphisms. Gene. 2004;338: 143–156. doi:10.1016/j.gene.2004.05.014

Supporting information

S1 Fig. Electrophoresis visualisation of *FokI* restriction enzyme analysis. bp, base pair; M, GeneRuler^{TM} DNA ladder

Fig 1 Click here to access/download;Figure;Fig 1.pdf

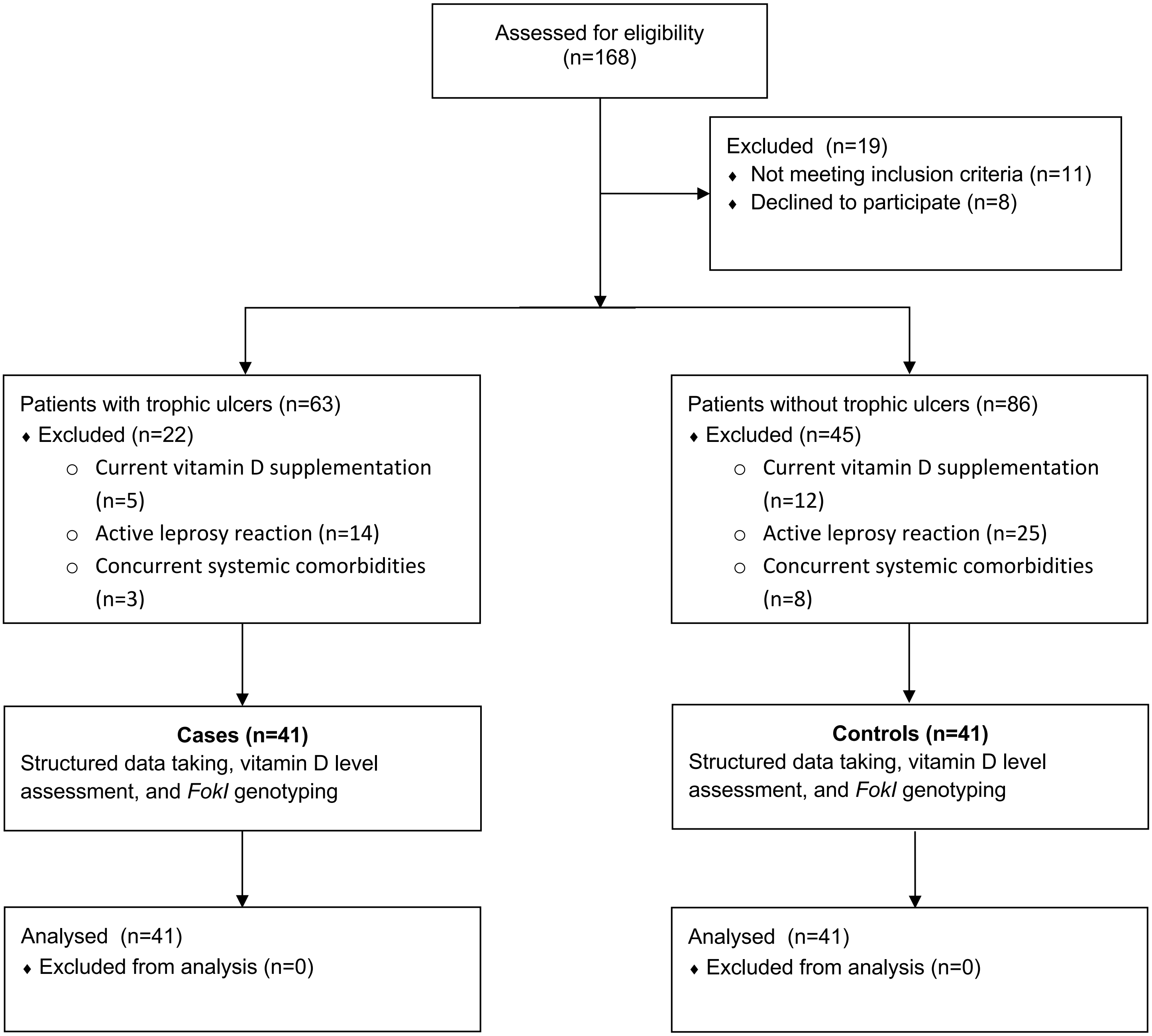

Fig 2

Click here to access/download;Figure;Fig 2.png

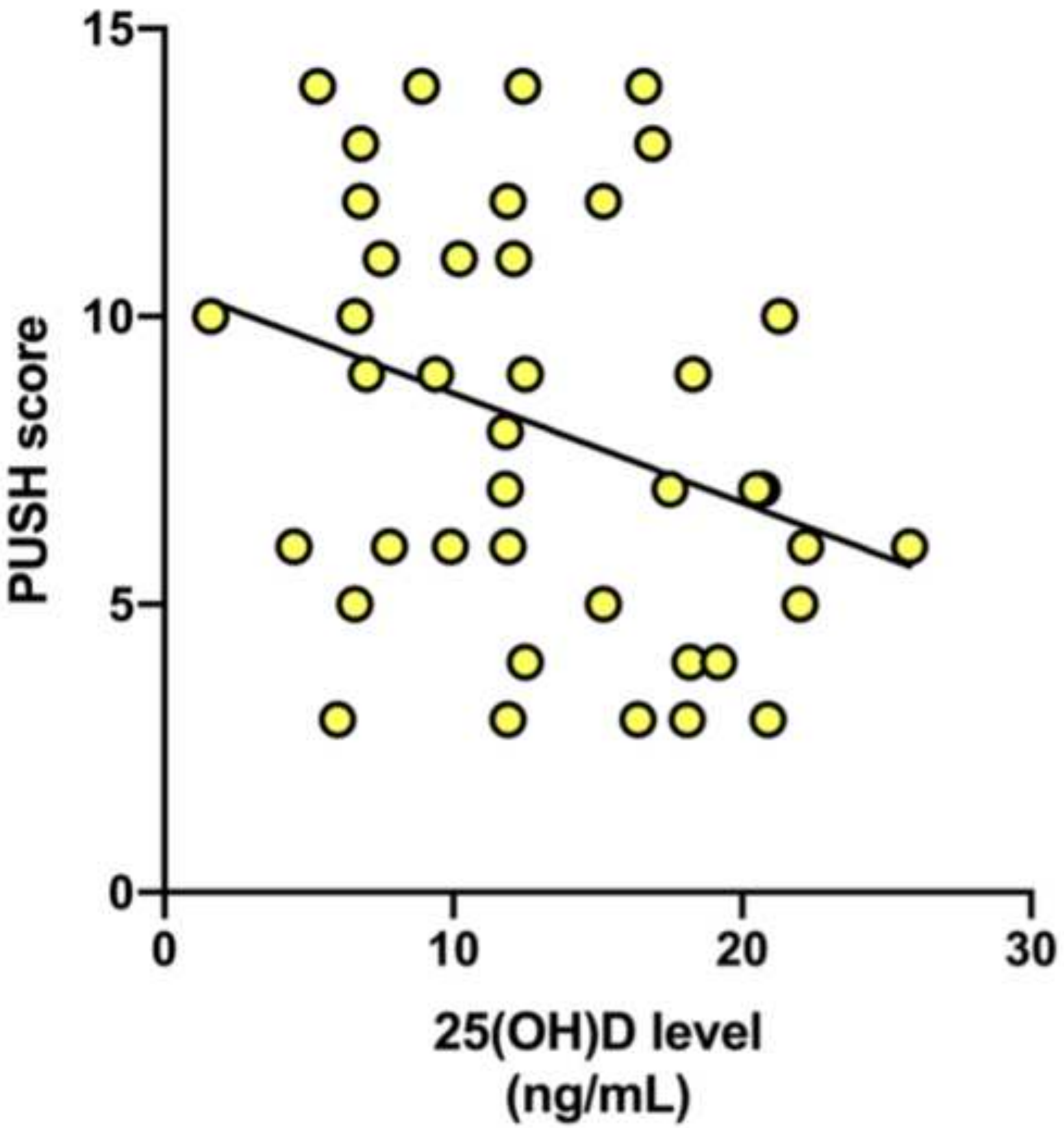

S1 Fig

Click here to access/download
Supporting Information
S1 Fig.pdf

Click here to access/download
Supporting Information
STROBE Checklist.pdf

Click here to access/download

Supporting Information

Figshare link.docx

F1000Research F1000Research 2025, 14:57 Last updated: 24 DEC 2025

Check for updates

STUDY PROTOCOL

REVISED Comparison of serum vitamin D level and vitamin D receptor gene *FokI* polymorphism in leprosy patients with and without trophic ulcers: protocol for a case-control study

[version 3; peer review: 3 approved with reservations]

Paulus Anthony Halim[1], Sondang P. Sirait[1], Eliza Miranda[1], Yulia Ariani[2,3], Wresti Indriatmi[1], Hok Bing Thio[4]

[1]Department of Dermatology and Venereology, Faculty of Medicine Universitas Indonesia, Dr. Cipto Mangunkusumo Hospital, Jakarta, 10430, Indonesia
[2]Department of Biology, Faculty of Medicine, Universitas Indonesia, Jakarta, 10430, Indonesia
[3]Human Genetic Research Center, Indonesian Medical Education and Research Institute (IMERI), Universitas Indonesia, Jakarta, 10430, Indonesia
[4]Department of Dermatology, Erasmus University Medical Center, Rotterdam, The Netherlands

v3 **First published:** 09 Jan 2025, **14**:57
https://doi.org/10.12688/f1000research.157869.1
Second version: 05 Sep 2025, **14**:57
https://doi.org/10.12688/f1000research.157869.2
Latest published: 23 Oct 2025, **14**:57
https://doi.org/10.12688/f1000research.157869.3

Abstract

Background

Leprosy, or Hansen's disease, is a chronic infectious disease that primarily affects the skin and peripheral nervous system, often leading to disabilities, including trophic ulcers (TU). The role of vitamin D and vitamin D receptor (*VDR*) gene polymorphisms in the development of TU among leprosy patients remain unclear.

Objective

This study aims to investigate the differences in serum 25-hydroxyvitamin D [25(OH)D] levels and the frequency of *VDR* gene *FokI* polymorphism between leprosy patients with and without TU, and to evaluate their association with the occurrence of TU.

Methods

This Institutional Review Board-approved, single-center, observational, analytic case-control study will enroll adult leprosy patients from the Dermatology and Venereology Clinic at Dr. Cipto

Open Peer Review

Approval Status ? ? ?

	1	2	3
**version 3** (revision) 23 Oct 2025			? view
**version 2** (revision) 05 Sep 2025		? view	
**version 1** 09 Jan 2025	? view		

1. **Khairuddin Djawad**, Hasanuddin University (Ringgold ID: 64739), Makassar, Indonesia
Wahidin Sudirohusodo General Hospital, Makassar, Indonesia

2. **Ravindra Turankar**, The Leprosy Mission Trust India, New Delhi, India

3. **ANABELL ALVARADO NAVARRO**, Centro Universitario de Ciencias de la Salud,

Mangunkusumo Hospital in Jakarta, Indonesia. Participants will be divided into cases (patients with TU) and controls (patients without TU). Serum 25(OH)D levels will be measured using a chemiluminescence immunoassay, and *FokI* polymorphism detection is conducted through polymerase chain reaction-restriction fragment length polymorphism (PCR-RFLP) methods. A total of 82 participants (41 cases and 41 controls) will be recruited. Statistical analysis will be performed to evaluate the differences and associations.

Results

Data collection commenced in March 2024 and was completed in September 2024, during the Indonesia's dry season. Data analysis and reporting are expected to be finalized by January 2025.

Discussion

The findings of this study will enhance the current understanding of the role of the vitamin D axis in the development of TU among leprosy patients. This could lead to new therapeutic strategies, such as vitamin D supplementation or personalized medicine based on genetic profiling, potentially reducing the burden of TU among leprosy patients.

Keywords
trophic ulcer, leprosy, vitamin D, vitamin D receptor gene polymorphisms

This article is included in the Genomics and Genetics gateway.

This article is included in the Neglected Tropical Diseases collection.

Universidad de Guadalajara, Guadalajara, Mexico

Any reports and responses or comments on the article can be found at the end of the article.

Corresponding authors: Sondang P. Sirait (sondang_dr@yahoo.com), Eliza Miranda (mirandaeliza74@gmail.com)

Author roles: Halim PA: Conceptualization, Funding Acquisition, Methodology, Writing – Original Draft Preparation, Writing – Review & Editing; **Sirait SP**: Conceptualization, Funding Acquisition, Methodology, Supervision, Writing – Review & Editing; **Miranda E**: Conceptualization, Methodology, Supervision, Writing – Review & Editing; **Ariani Y**: Conceptualization, Methodology, Supervision; **Indriatmi W**: Methodology, Writing – Review & Editing; **Thio HB**: Funding Acquisition, Methodology, Supervision, Writing – Review & Editing

Competing interests: No competing interests were disclosed.

Grant information: The study is funded by the Directorate of Research and Development, Universitas Indonesia under the Hibah PUTI 2023 scheme (Grant No. NKB-689/UN2.RST/HKP.05.00/2023).
The funders had no role in study design, data collection and analysis, decision to publish, or preparation of the manuscript.

Copyright: © 2025 Halim PA *et al*. This is an open access article distributed under the terms of the Creative Commons Attribution License , which permits unrestricted use, distribution, and reproduction in any medium, provided the original work is properly cited.

How to cite this article: Halim PA, Sirait SP, Miranda E *et al.* **Comparison of serum vitamin D level and vitamin D receptor gene *FokI* polymorphism in leprosy patients with and without trophic ulcers: protocol for a case-control study [version 3; peer review: 3 approved with reservations]** F1000Research 2025, **14**:57 https://doi.org/10.12688/f1000research.157869.3

First published: 09 Jan 2025, **14**:57 https://doi.org/10.12688/f1000research.157869.1

REVISED **Amendments from Version 2**

In response to Reviewer 2's comments, we revised the manuscript to improve clarity and methodological transparency. We detailed how potential confounding variables such as neuropathy, deformities, nutritional status, and sun exposure are assessed and included in multivariate analysis. We clarified that participant recruitment was conducted during the Indonesian dry season (March to September 2024) to minimize variability in vitamin D levels. The rationale for using PCR-RFLP for genotyping was explained based on feasibility, simplicity of the *FokI* polymorphism, and cost-effectiveness. We acknowledged the potentially optimistic sample size for the *FokI* polymorphism analysis assumption as a limitation in the discussion. Ulcer documentation procedures were strengthened by clarifying that ulcer assessments were conducted by a senior dermatology resident under consultant dermatologist supervision using standardized PUSH criteria, supported by clinical photographs for verification. Ethical safeguards for image capture and storage were described. Additional minor revisions included stating the planned sample size in the abstract, adding gene-environment interaction to the objectives, outlining missing data handling strategies, and clarifying covariate selection criteria. A note was also added that a participant flow diagram will be included in the future publication of study results. We added a paragraph of study limitations on the Discussion section.

Any further responses from the reviewers can be found at the end of the article

Introduction

Background

Leprosy, also known as Hansen's disease (HD), is a chronic infectious disease caused by the bacteria *Mycobacterium leprae* and *Mycobacterium lepromatosis*. This disease primarily affects the skin and peripheral nervous system, leading to disabilities. Leprosy remains endemic in many parts of the world, with Brazil, India, and Indonesia collectively accounting for 80% of new cases globally.[1] In 2019, the Indonesian Ministry of Health reported 17,439 new cases of HD, while in 2020, only 11,173 new cases were identified.[2,3] The COVID-19 pandemic has impacted early detection efforts due to social restrictions and the reallocation of healthcare resources, potentially increasing the incidence of disabilities resulting from delayed diagnosis and treatment.[4]

Peripheral nerve damage secondary to leprosy can lead to various types of disabilities. Ulcers are a common complication and are classified as grade 2 disabilities.[5] The rate of grade 2 disabilities is a key indicator in the World Health Organization's (WHO) 2021-2030 leprosy eradication strategy. In 2021, the rate of grade 2 disabilities in Indonesia was 2.48 cases per 1,000,000 people, whereas the WHO's 2030 target is 0.12 cases per 1,000,000 people.[2] Disabilities associated with leprosy create social stigma and lead to significant economic losses, including healthcare costs and reduced productivity.[6]

Ulcers occur in 10–20% of HD patients and are mostly classified as trophic ulcers (TU).[5] Most ulcers in HD patients occur in the lower extremities, particularly on the plantar surface of the feet.[5,7] Static and dynamic deformities of the feet, coupled with reduced sensitivity, lead to recurring wounds that do not heal and become chronic ulcers.[5,7] The occurrence of TU in leprosy patients can be influenced by several factors, including neuropathy, physical deformities, nutritional deficiencies, and other systemic comorbidities. In leprosy patients, ulcers are often difficult to treat and frequently recur. If not properly managed, ulcers can become secondarily infected and may even undergo malignant transformation.[8]

Vitamin D is a steroid hormone that plays a crucial role as an immune system modulator.[9,10] Over the past decade, studies have reported the role of vitamin D in wound healing.[9,11–13] Vitamin D enhances the differentiation of fibroblasts and keratinocytes, mainly through the modulation of growth factors and cytokine production.[14,15] In vivo, vitamin D is converted to its active form, 25-hydroxyvitamin D [25(OH)D], by the hepatic hydroxylase enzymes, which is an indicator of vitamin D status. In the innate immune system, 25(OH)D can trigger the production of antimicrobial peptides (AMPs) by monocytes and macrophages.[10] Additionally, 25(OH)D reduces the production of proinflammatory cytokines and increases anti-inflammatory responses.[16] The kidneys or injured tissues can convert 25(OH)D into 1,25-dihydroxy vitamin D3 or calcitriol, which regulates the expression of cathelicidin and defensins.[15,17] These AMPs can kill intracellular bacteria and play a significant role in wound healing.[10,15]

The biological effects of vitamin D are mediated by the vitamin D receptor (VDR), which is encoded by the *VDR* gene on chromosome 12. Animal studies have reported that loss of *VDR* inhibits tissue re-epithelialization and inflammatory response in wound healing.[18,19] Various single nucleotide polymorphisms (SNPs) can affect the structure and function of VDR. The *FokI* polymorphism (rs2228570), which involves a nucleotide substitution from thymine (T) to cytosine (C) at the start codon of the *VDR* gene, is the only polymorphism known to structurally alter the VDR protein. The mutant T allele (*f* allele) yields a receptor with a longer amino acid chain compared to the wild-type C allele (*F* allele).[20] The *F* allele produces a VDR with more optimal gene transcription effects, thereby triggering increased immune responses and wound

Table 1. Primary and secondary objectives.

Objective type	Description
Primary	• To analyze the differences of 25-hydroxyvitamin D [25(OH)D] levels in leprosy patients with and without trophic ulcers.<br>• To analyze the proportion difference of *FokI* polymorphism in leprosy patients with and without trophic ulcers.<br>• To evaluate whether 25(OH)D levels and *FokI* polymorphism are associated with the occurrence of trophic ulcers in leprosy patients.
Secondary	To determine whether there is any correlation between 25(OH)D levels and the severity of trophic ulcers.

healing.[20,21] A study in Medan, Indonesia reported that the frequency of homozygous mutant subjects for the *FokI* polymorphism was 19.2% in multibacillary HD patients and 13.7% in healthy household contacts of HD patients.[22]

Previous studies have shown that serum 25(OH)D levels in patients with other chronic ulcers (e.g., venous or diabetic ulcers) are decreased,[23,24] and vitamin D supplementation can improve wound healing in these conditions.[12,13] The role of vitamin D in leprosy is increasingly investigated, with previous studies reporting varied results.[25–27] However, to the best of our knowledge, no research has compared vitamin D levels and the polymorphisms of its receptor in leprosy patients with and without TU.

Aim
The aim of this study is to investigate whether vitamin D and the polymorphisms of its receptor (*VDR*) play a role in the development of TU in leprosy patients. The primary and secondary objectives of the study are displayed in Table 1. An exploratory objective is to assess potential interactions between serum vitamin D levels and *FokI* polymorphism in relation to TU development.

Protocol
Study design
This study is a single-center, observational, analytic case-control study.

Site and population
Leprosy patients with and without TU who are attending the Dermatology and Venereology Clinic at Dr. Cipto Mangunkusumo Hospital in Jakarta, Indonesia will be recruited. Serum 25(OH)D levels will be analyzed at the Clinical Pathology Laboratory of the same hospital. Examination of the *FokI* polymorphism will be conducted at the Laboratory of the Medical Biology Department, Faculty of Medicine, Universitas Indonesia, Jakarta.

Case and control definitions
Both cases and controls include adult leprosy patients diagnosed according to the cardinal signs defined by WHO. Patients who are newly diagnosed, currently undergoing treatment, or have been released from treatment are eligible for inclusion. The inclusion and exclusion criteria are detailed in Table 2. Screening for excluded systemic conditions was conducted through structured clinical interviews and review of the patients' medical records, including relevant laboratory and radiologic data when available. The control group will consist of leprosy patients without TU, who meet the same inclusion and exclusion criteria as the case group.

Subject recruitment
Subjects will be recruited consecutively based on the eligibility criteria. All leprosy patients attending the Dermatology and Venereology Clinic of Dr. Cipto Mangunkusumo Hospital will be given the opportunity to participate in this study and will be screened according to the inclusion and exclusion criteria. Participant recruitment is conducted during Indonesia's dry season (March to September 2024). Before further examination, each potential subject will be informed about the purpose and methods of the study, as well as the benefits and potential disadvantages of participating. Written informed consent will be obtained from each subject. A participant flow diagram will be included during reporting of study results.

Table 2. Inclusion and exclusion criteria.

Criteria	Description
Inclusion	• Patients willing to participate in the study by completing and signing an informed consent form.<br>• Male or female patients aged ≥18 years old.<br>• Patients who meet the World Health Organization (WHO) diagnostic criteria for leprosy, including those who are newly diagnosed, currently undergoing treatment, or have been released from treatment.<br>• Leprosy patients with trophic ulcers (case group), adhering to the following criteria:<br>○ Ulcer duration of ≥4 weeks, with no history of sharp debridement or callus thinning during this period.<br>○ Wound depth not exceeding the subcutaneous layer.<br>○ Ulcer not secondarily infected or only mildly infected (characterized by erythema <2 cm around the ulcer, potential signs of edema, pain, minimal purulent secretions, or warmth on palpation).
Exclusion	• Patients who have consumed vitamin D supplementation within the past 4 weeks.<br>• Patients exhibiting signs and symptoms of leprosy reactions.<br>• Patients with concurrent systemic infections, diabetes mellitus, severe liver dysfunction, end-stage kidney disease, malignancy, and autoimmune diseases.

Procedures and examinations

History taking and physical examination will be conducted in a structured manner using a standardized study data collection form. Anamnesis will include identity, sociodemographic information (e.g., education level, occupation, and household income), leprosy history (duration, treatment, and reaction history), sun exposure duration, smoking status, and ulcer history. Sun exposure will be estimated based on the number of hours spent outdoors per week as reported by the subject.

Clinical data related to leprosy, including disease duration, WHO and Ridley-Jopling classification, treatment status, and history of reactions, will be obtained through patient interviews and verified from medical records where available. Physical examination will include vital signs, anthropometric measurements, dermatologic status of the ulcer, presence of non-ulcer deformities (e.g., claw hand, foot drop, amputation). Sensory function will be evaluated using Semmes-Weinstein monofilaments (0.2 g for hands, 2 g for feet) to clinically assess peripheral neuropathy. Nutritional status will be assessed using body mass index (BMI), and categorized based on the Asia-Pacific classification.[28]

Clinical photographs of the subjects' hands and feet are collected, clearly showing the ulcers if present. If ulcers occur on other areas of the extremities (e.g., knee or elbow), documentation will also be performed on these areas. Photos are taken using an iPhone 12 camera (12 megapixel, aperture size F1.6, focal length 26 mm, sensor size 1/2.55", pixel size 1.7 μm) from a distance of 15 cm. A blue cloth will be used as the background during photo taking.

The dimensions of the ulcer are measured using a digital caliper, with the ulcer area calculated by the multiplying the largest vertical and horizontal lengths that intersect perpendicularly. Ulcer depth was assessed based on clinical examination of the wound bed and further categorized using the Pressure Ulcer Scoring System (grades I to III). In cases where necrotic tissue or slough prevented adequate visualization, sharp debridement was performed as part of standard clinical care to facilitate accurate depth assessment. Only patients with ulcers confined to the subcutaneous layer were included, while those with wounds exposing deeper structures such as fascia, tendon, or bone were excluded.

Ulcer severity will be assessed using the Pressure Ulcer Scale for Healing (PUSH) criteria, which encompasses ulcer area, exudate amount, and wound tissue type (Table 3). This scoring system is chosen for its validated and standardized criteria. All ulcer assessments are performed by the first author (PAH), who at time of the study is a third-year dermatology resident, and directly supervised by two board-certified consultant dermatologists (SPS and EM) to ensure consistency and reduce inter-observer variability. Clinical photographs of each ulcer will be reviewed for retrospective verification, if required. In patients with multiple ulcers, the largest ulcer by area will be selected for reporting and further statistical analysis.

Table 3. Pressure Ulcer Scale for Healing (PUSH).

Characteristics	Score						Subscore
**Length × Width** (in cm^2)	**0** 0	**1** <0.3	**2** 0.3–0.6	**3** 0.7–1.0	**4** 1.1–2.0	**5** 2.1–3.0	
		**6** 3.1–4.0	**7** 4.1–8.0	**8** 8.1–12.0	**9** 12.1–24.0	**10** >24.0	
**Exudate amount**	**0** None	**1** Light	**2** Moderate	**3** Heavy			
**Tissue type**	**0** Closed	**1** Epithelial Tissue	**2** Granulation Tissue	**3** Slough	**4** Necrotic tissue		
						**Total:**	

Adapted from Gardner et al.[32]

Laboratory prodecures

Blood samples totaling 6 mL will be collected from each subjects into plain (red-capped) and K_3 EDTA (purple-capped) tubes and temporarily stored in a cool box at 2–8°C. The blood samples will be sent to the laboratories within 2 hours of collection.

25(OH)D Level quantification

Blood collected in a plain tube will be analyzed for 25(OH)D levels using the Architect 25(OH)D vitamin kit (Abbott Diagnostics, Lake Forest, IL, USA) based on the chemiluminescence immunoassay method. Vitamin D status will be classified under four categories: severe deficiency (<10 ng/mL), deficiency (10-20 ng/mL), insufficiency (20-30 ng/mL), and sufficiency (>30 ng/mL).[29]

FokI polymorphism detection

The anticoagulated blood will be used for *FokI* polymorphism detection, which consists of DNA extraction, subsequent amplification using polymerase chain reaction (PCR), and polymorphism detection using the restriction fragment length polymorphism (RFLP) method. DNA extraction from EDTA blood samples will be performed using the salting out method.[30] Extracted genetic materials will be stored on a -80°C freezer for subsequent analysis.

Amplification of the genetic materials are performed using PCR. The primers for the reaction are based on a previous study by Soroush et al.,[20] with a forward primer 5′-CACTGACTCTGGCTCTGACCGT-3′ and a reverse primer 5′-AACACCTTGCTTCTTCTCCCTCC-3′. Initial denaturation of the genetic material will be performed at 95°C for 5 minutes. Amplification will be conducted for 35 cycles with denaturation at 95°C for 30 seconds, annealing at 60°C for 30 seconds, and extension at 72°C for 30 seconds. The final extension will be performed at 72°C for 7 minutes, resulting in a nucleic acid product of 250 base pairs (bp) in length.

The RFLP will be conducted using the *FokI* restriction enzyme (New England Biolabs, Ipwich, MA, USA). After incubation, the results will be visualized with 2% agarose gel electrophoresis. The genetic material fractions that will be detected are 192 and 58 bp in homozygous *ff* genotype samples, 250 bp, 192 bp, and 58 bp in heterozygous *Ff* genotype samples, and an intact 250 bp fragment in *FF* genotype samples.

Sample size estimates

The calculation of sample size is carried out using a type I error probability of 0.05 and type II error of 0.20, corresponding to a power of 80%.

To test the difference in mean 25(OH)D levels, sample size calculation for the difference between the mean values of two independent groups is performed using the following formula[31]:

$$n_1 = n_2 = 2\left[\frac{(z_\alpha + z_\beta)s}{(x_1 - x_2)}\right]^2$$

where n_1 = n_2 = minimum sample size for each group; z_α = z-score corresponding to the type I error probability, which is 1.96; $z_\beta = z$-score corresponding to the type II error probability ($\beta = 0.20$), which is 0.84); s = standard deviation;

and $(x_1 - x_2)$ = clinically significant mean difference. The clinically significant mean difference is determined to be 5 ng/mL. Since there has been no studies reporting the difference in 25(OH)D levels between leprosy groups with and without TU, the standard deviation value used is based on a previous study in general leprosy patients, which is 5.42 ng/mL.[27] Thus, the sample size needed to detect a difference in 25(OH)D levels in leprosy patients with and without TU is 19 per group.

To test the effect of *FokI* polymorphism on the occurrence of TU in leprosy patients, the sample size for an unmatched case-control study is calculated using the formula[31]:

$$n_1 = n_2 = \frac{\left(z_\alpha\sqrt{2PQ} + z_\beta\sqrt{P_1Q_1 + P_2Q_2}\right)^2}{(P_1 - P_2)^2}$$

where $n_1 = n_2$ = minimum sample size for each group; $z_\alpha = 1.96$; $z_\beta = 0.84$; P_1 = estimated proportion of effect in the case group calculated using $P_1 = \frac{OR\times P_2}{(1-P_2)+(OR\times P_2)}$; OR = estimated odds ratio; P_2 = proportion of effect in the control group; $P = ½ (P_1 + P_2)$; and $Q = 1 – P$. The proportion of the homozygous *ff* genotype in leprosy patients based on a previous study by Lubis et al.[22] is 19.2%. Assuming an estimated odds ratio (OR) of 3.9 as the effect size of *FokI* polymorphism on TU occurrence in leprosy patients, the formula corresponds to a sample size of 41 for each of the case and control groups. While this assumption may be optimistic, it was deemed acceptable given the exploratory nature of this study and the limited prior data available in this population. The final analysis will interpret findings in light of this assumption, acknowledging that smaller true effect sizes may reduce the statistical power.

Finally, to test the correlation between 25(OH)D levels and ulcer severity, sample size calculation for correlation is conducted using the following formula[31]:

$$n = \left[\frac{\left(z_\propto + z_\beta\right)}{0.5\ln\left(\frac{1+r}{1-r}\right)}\right]^2 + 3$$

where n = minimum sample size for the group; $z_\alpha = 1.96$; $z_\beta = 0.84$; and r = estimated correlation coefficient. A moderate correlation (r = 0.5–0.7) is estimated between ulcer severity score and serum 25(OH)D levels. Thus, setting the correlation coefficient at 0.5, the minimal sample size is 29 for this analysis. The overall sample size for this study is determined based on the largest minimum sample size required to test all outcomes, which is 41 for each group of leprosy with and without TU, totaling 82 subjects.

Data analysis

All data obtained from anamnesis, physical examinations, and laboratory tests are recorded in the research file. The collected data will be reviewed, coded, and then entered into a master table in Excel version 16 (Microsoft Corporation, Washington, USA). Data analysis will be conducted using STATA version 16.0 (StataCorp LLC, College Station, TX, USA) and the online software SNPStats (www.snpstats.net, Catalan Institute of Oncology, Spain).

Sociodemographic and clinical characteristics data will be processed descriptively and presented in tables to understand the distribution of data across groups. Clinical characteristics of TU will also be described and presented in a table. Data normality will be tested using the Kolmogorov-Smirnov test. Numerical data will be presented as mean and standard deviation (SD), or as median and interquartile range (IQR) if not normally distributed.

A significance level of $p < 0.05$ will be used throughout the study. The comparison of means between two groups will be conducted using the independent t-test for normally distributed data or the Mann-Whitney U test if the data are not normally distributed. Relationship between two categorical variables will be analyzed using the chi-square or Fisher's exact test, as appropriate. Correlations between two numeric variables will be calculated using the Pearson's correlation coefficient for normally distributed data or Spearman's rank correlation coefficient if the data are not normally distributed.

The Hardy-Weinberg equilibrium will be assessed to determine if the observed genotype frequencies are consistent with those expected in a population for both case and control groups. Different genetic models (i.e. codominant, dominant. recessive, overdominant, and log-additive) for the *FokI* polymorphism will be evaluated. Both the Akaike information criterion (AIC) and the Bayesian information criterion (BIC) will be used to compare these models. The genetic model yielding the lowest AIC and BIC values will be considered the best-fitting model. As only one genetic variant (*FokI*) is analyzed, correction for multiple testing is not required.

Finally, multivariate logistic regression analyses will be conducted to identify factors independently associated with the occurrence of TU in leprosy patients. The variables may include vitamin D level, *FokI* genotype, and relevant sociodemographic and clinical factors such as neuropathy, non-ulcer deformities, smoking, and leprosy classification. Variables with a p-value < 0.25 in bivariate analysis will be considered for inclusion in the multivariate model. Adjusted odds ratios (ORs) and 95% confidence intervals (CIs) will be reported for the variables included in the final model.

As this is a prospective study, missing data are expected to be minimal. Any missing values will be transparently reported. Where applicable, sensitivity analyses will be performed using either complete-case analysis or multiple imputation methods to assess the robustness of the findings.

Ethics and consent

This study protocol has received ethical clearance from the Health Research Ethics Committee –Faculty of Medicine Universitas Indonesia and Dr. Cipto Mangunkusumo Hospital (HREC FMUI-CMH), with the approval letter number KET-82/UN2.F1/ETIK/PPM.00.02/2024, dated January 15, 2024.

We will explain to potential subjects that participation is voluntary and does not affect the management provided at the hospital. Written informed consent will be obtained from each subject. Clinical photographs will be taken only following written consent, primarily focusing on distal extremities where most ulcers occur. Any identifiable features, such as tattoos or nevi, will be digitally blurred. All image files will be stored on a password-protected USB drive accessible only to study investigators.

Dissemination

The results of the study will be disseminated through peer-reviewed publications and will be made available in an open-access format. Any significant changes to the protocol will be communicated directly to HREC FMUI-CMH. We also plan to publish any modifications made to this protocol during publication of the study results.

Study status

Subject recruitment, assessment, and laboratory work for 25(OH)D level measurement and *FokI* polymorphism detection have been completed. Formal data analysis is currently ongoing.

Discussion

Summary

This study aims to investigate the association between serum vitamin D levels and *FokI* polymorphism of *VDR* in leprosy patients, comparing those with and without TU. Leprosy often leads to peripheral nerve damage, with TU as a common complication. We hypothesize that vitamin D and its receptor polymorphisms, particularly *FokI*, might influence ulcer development in these patients.

Our study is a single-center, case-control study involving adult leprosy patients with and without TU. Serum vitamin D levels are quantified using chemiluminescence immunoassay, while the *FokI* polymorphism of the *VDR* gene is detected through PCR-RFLP method. The primary objectives are to analyze differences in 25(OH)D levels and the proportion of *FokI* polymorphism between the two groups, and to evaluate their association with TU occurrence. Secondary objectives include examining the correlation between vitamin D level and the severity of TU.

In this study, we utilize the PCR-RFLP method to detect the *FokI* polymorphism. While newer techniques such as TaqMan assays and next generation sequencing offer higher throughput and potentially greater precision, PCR-RFLP remains a widely accepted method for analysing well-characterized SNP with known restriction sites. Furthermore, given that only one SNP (*FokI*) is being investigated in this study, the use of multiplex or sequencing platforms was not warranted. PCR-RFLP was selected based on considerations of cost-effectiveness, simplicity, and compatibility with the technical capacity of our local laboratory setting.

Perspective and clinical implications

Trophic ulcers may still occur in leprosy patients long after treatment completion. As the world approaches the leprosy post-elimination era, TU will continue to cause significant morbidity. Therefore, the study's focus on the potential influence of vitamin D and its receptor polymorphisms on TU development in leprosy is of significant clinical importance.

If substantial evidence is found, this could pave the way for new treatment strategies, such as vitamin D supplementation or personalized therapies based on genetic profiles in leprosy patients. This could enhance patient outcomes, reduce the incidence of trophic ulcers, and consequently diminish the healthcare and social burdens associated with leprosy-related disabilities. Additionally, the findings could contribute to a better understanding of the role of the vitamin D axis in the pathophysiology of TU and other chronic ulcers in general.

Limitations

This study has several limitations. First, the single-center design may limit generalizability to broader populations. Second, the observational nature of a case–control study restricts the ability to infer causality. Third, although multivariable analyses will be conducted, the potential for residual confounding remains. Finally, the sample size was calculated based on an estimated odds ratio that may be optimistic due to limited prior data on the association between *FokI* polymorphism and trophic ulcer risk in leprosy. As such, the findings will be interpreted with caution and should be validated in larger, multi-center studies.

Data availability

Underlying data

No data is associated with this article.

Reporting guidelines

The final publication of the study results will adhere to the Strengthening the Reporting of Observational Studies in Epidemiology (STROBE) statement.

References

1. World Health Organization: **Weekly epidemiological report (WER) 10 September 2021.** *Weekly Epidemiological Report.* 2021; **96**(36): 421–444.
2. Kementerian Kesehatan Republik Indonesia: *Profil Kesehatan Indonesia Tahun 2020.* Kementerian Kesehatan RI; 2021.
3. Kementerian Kesehatan Republik Indonesia: *Profil Kesehatan Indonesia Tahun 2021.* Kementerian Kesehatan RI; 2022.
4. Chen X, Liu HB, Shui TJ, *et al.*: **Risk factors for physical disability in patients with leprosy disease in Yunnan, China: Evidence from a retrospective observational study.** *PLoS Negl. Trop. Dis.* 2021; **15**(11): e0009923.
PubMed Abstract | Publisher Full Text | Free Full Text
5. Upputuri B, Srikantam A, Mamidi RS: **Comorbidities associated with non-healing of plantar ulcers in leprosy patients.** *PLoS Negl. Trop. Dis.* 2020; **14**(6): e0008393–e0008312.
PubMed Abstract | Publisher Full Text | Free Full Text
6. Xiong M, Li M, Zheng D, *et al.*: **Evaluation of the economic burden of leprosy among migrant and resident patients in Guangdong Province, China.** *BMC Infect. Dis.* 2017; **17**(1): 760.
PubMed Abstract | Publisher Full Text | Free Full Text
7. Barreto JG, Salgado CG: **Clinic-epidemiological evaluation of ulcers in patients with leprosy sequelae and the effect of low level laser therapy on wound healing: A randomized clinical trial.** *BMC Infect. Dis.* 2010; **10**: 237.
PubMed Abstract | Publisher Full Text | Free Full Text
8. Karthikeyan K, Thappa DM: **Squamous cell carcinoma in plantar ulcers in leprosy: a study of 11 cases.** *Indian J. Lepr.* 2003; **75**(3): 219–224.
PubMed Abstract
9. Bikle D, Christakos S: **New aspects of vitamin D metabolism and action — addressing the skin as source and target.** *Nat. Rev. Endocrinol.* 2020; **16**(4): 234–252.
PubMed Abstract | Publisher Full Text
10. Schwalfenberg GK: **A review of the critical role of vitamin D in the functioning of the immune system and the clinical implications of vitamin D deficiency.** *Mol. Nutr. Food Res.* 2011; **55**(1): 96–108.
PubMed Abstract | Publisher Full Text
11. Razzaghi R, Pourbagheri H, Momen-Heravi M, *et al.*: **The effects of vitamin D supplementation on wound healing and metabolic status in patients with diabetic foot ulcer: A randomized, double-blind, placebo-controlled trial.** *J. Diabetes Complicat.* 2017; **31**(4): 766–772.
PubMed Abstract | Publisher Full Text
12. Burkiewicz CJCC, Guadagnin FA, Skare TL, *et al.*: **Vitamin D and skin repair: a prospective, double-blind and placebo controlled study in the healing of leg ulcers.** *Rev. Col. Bras. Cir.* 2012; **39**(5): 401–407.
PubMed Abstract | Publisher Full Text
13. Dai J, Yu M, Chen H, *et al.*: **Association between serum 25-OH-vitamin D and diabetic foot ulcer in patients with type 2 diabetes.** *Front. Nutr.* 2020; **7**: 7.
PubMed Abstract | Publisher Full Text | Free Full Text
14. Ding J, Kwan P, Ma Z, *et al.*: **Synergistic effect of vitamin D and low concentration of transforming growth factor beta 1, a potential role in dermal wound healing.** *Burns.* 2016; **42**(6): 1277–1286.
PubMed Abstract | Publisher Full Text
15. Oda Y, Tu CL, Menendez A, *et al.*: **Vitamin D and calcium regulation of epidermal wound healing.** *J. Steroid Biochem. Mol. Biol.* 2016; **164**: 379–385.
PubMed Abstract | Publisher Full Text | Free Full Text
16. Afarideh M, Ghanbari P, Noshad S, *et al.*: **Raised serum 25-hydroxyvitamin D levels in patients with active diabetic foot ulcers.** *Br. J. Nutr.* 2016; **115**(11): 1938–1946.
PubMed Abstract | Publisher Full Text
17. Segaert S: **Vitamin D regulation of cathelicidin in the skin: Toward a renaissance of vitamin D in dermatology?** *J. Invest. Dermatol.* 2008; **128**(4): 773–775.
PubMed Abstract | Publisher Full Text
18. Oda Y, Hu L, Nguyen T, *et al.*: **Vitamin D receptor is required for proliferation, migration, and differentiation of epidermal stem cells and progeny during cutaneous wound repair.** *J. Invest. Dermatol.* 2018; **138**(11): 2423–2431.
PubMed Abstract | Publisher Full Text | Free Full Text
19. Song L, Papaioannou G, Zhao H, *et al.*: **The Vitamin D receptor regulates tissue resident macrophage response to injury.** *Endocrinology.* 2016; **157**(10): 4066–4075.
PubMed Abstract | Publisher Full Text | Free Full Text
20. Soroush N, Radfar M, Hamidi AK, *et al.*: **Vitamin D receptor gene *FokI* variant in diabetic foot ulcer and its relation with oxidative stress.** *Gene.* 2017; **599**: 87–91.
PubMed Abstract | Publisher Full Text
21. Usategui-Martín R, De Luis-Román DA, Fernández-Gómez JM, *et al.*: **Vitamin D Receptor (*VDR*) gene polymorphisms modify the response to vitamin D supplementation: A systematic review and meta-analysis.** *Nutrients.* 2022; **14**(2).
PubMed Abstract | Publisher Full Text | Free Full Text
22. Lubis RD, Roesyanto-Mahadi ID, Siregar Y, *et al.*: ***FokI* Vitamin D receptor gene polymorphism in leprosy household contacts.** *Malay J. Med. Health Sci.* 2021; **17**: 113–116.
23. Priyanto MH, Legiawati L, Saldi SRF, *et al.*: **Comparison of vitamin D levels in diabetes mellitus patients with and without diabetic

foot ulcers: An analytical observational study in Jakarta, Indonesia. *Int. Wound J.* 2023; **20**: 2028–2036.
PubMed Abstract | Publisher Full Text | Free Full Text

24. Agius C, Micallef D, Brincat I, *et al.*: **Plasma total ascorbic acid and serum 25-hydroxy-vitamin-D status in patients with venous leg ulcers: a case–control study.** *Int. J. Lower Extremities Wound.* 2021; **23**: 421–427.
PubMed Abstract | Publisher Full Text

25. Luong KVQ, Nguyen LTH: **Role of the vitamin D in leprosy.** *Am. J. Med. Sci.* 2012; **343**(6): 471–482.
PubMed Abstract | Publisher Full Text

26. Dennison CL, de Oliveira LB, Fraga LA d O, *et al.*: **Mycobacterium leprae–helminth co-infections and vitamin D deficiency as potential risk factors for leprosy: A case–control study in south-eastern Brazil.** *Int. J. Infect. Dis.* 2021; **105**: 261–266.
PubMed Abstract | Publisher Full Text

27. Darus NIM, Lubis RD, Jusuf NK: **Analysis of serum vitamin D level in leprosy patients.** *Bali Med. J.* 2019; **8**(3): 795–799.
Publisher Full Text

28. Pan WH, Yeh WT: **How to define obesity? Evidence-based multiple action points for public awareness, screening, and treatment: an extension of Asian-Pacific recommendations.** *Asia Pac. J. Clin. Nutr.* 2008; **17**(3): 370–374.
PubMed Abstract

29. Avci E, Demir S, Aslan D, *et al.*: **Assessment of Abbott Architect 25-OH vitamin D assay in different levels of vitamin D.** *J. Med. Biochem.* 2020; **39**(1): 100–107.
PubMed Abstract | Publisher Full Text | Free Full Text

30. Miller SA, Dykes DD, Polesky HF: **A simple salting out procedure for extracting DNA from human nucleated cells.** *Nucleic Acids Res.* 1988; **16**(3): 1215.
PubMed Abstract | Publisher Full Text | Free Full Text

31. Madiyono B, Sastroasmoro S, Budiman I, *et al.*: **Perkiraan besar sampel.**Sastroasmoro S, Ismael S, editors. *Dasar-Dasar Metodologi Penelitian Klinis.* 5th ed. Sagung Seto; 2014; pp. 352–387.

32. Gardner SE, Frantz RA, Bergquist S, *et al.*: **A prospective study of the pressure ulcer scale for healing (PUSH).** *J. Gerontol.* 2005; **60**(1): 93–97.
Publisher Full Text

Open Peer Review

Current Peer Review Status: ? ? ?

Version 3

Reviewer Report 25 November 2025

https://doi.org/10.5256/f1000research.189764.r427045

© 2025 NAVARRO A. This is an open access peer review report distributed under the terms of the Creative Commons Attribution License, which permits unrestricted use, distribution, and reproduction in any medium, provided the original work is properly cited.

?

ANABELL ALVARADO NAVARRO
Centro Universitario de Ciencias de la Salud, Universidad de Guadalajara, Guadalajara, Mexico

The article addresses a health problem that has been considered a disease of little interest for study. In addition, the authors responded to comments and suggestions made by other reviewers, so, in my opinion, the manuscript is complete and scientifically sound.
However, it is not clear to me why the results have not been included, given that the protocol mentions that the study subjects were recruited in September 2024. However, the results are not reported.
In addition, several sections are written in the future tense.
.

Is the rationale for, and objectives of, the study clearly described?
Yes

Is the study design appropriate for the research question?
Yes

Are sufficient details of the methods provided to allow replication by others?
Yes

Are the datasets clearly presented in a useable and accessible format?
No

Competing Interests: No competing interests were disclosed.

Reviewer Expertise: autoimmunity, immunogenetics, inflammation, immunology

I confirm that I have read this submission and believe that I have an appropriate level of expertise to confirm that it is of an acceptable scientific standard, however I have significant reservations, as outlined above.

Version 2

Reviewer Report 07 October 2025

https://doi.org/10.5256/f1000research.187546.r415296

© 2025 Turankar R. This is an open access peer review report distributed under the terms of the Creative Commons Attribution License, which permits unrestricted use, distribution, and reproduction in any medium, provided the original work is properly cited.

?

Ravindra Turankar
[1] Stanley Browne Research Laboratory, The Leprosy Mission Trust India, New Delhi, New Delhi, India
[2] Stanley Browne Research Laboratory, The Leprosy Mission Trust India, New Delhi, New Delhi, India

COMMENTS ON THE STUDY PROTOCOL ENTITLED, "Comparison of serum vitamin D level and vitamin D receptor gene *FokI* polymorphism in leprosy patients with and without trophic ulcers: protocol for a case-control study".

This is a well-designed and relevant protocol that explores vitamin D status and the VDR FokI polymorphism in leprosy patients with and without trophic ulcers. The study addresses a significant gap with potential implications for clinical management and public health. I have a few points for clarification and improvement.
The following points are being made as follows:

Major Comments:

1. Confounders:
    - The multifaceted factors leading to ulceration in leprosy encompass neuropathy, deformities, nutrition, footwear, and sun exposure. It is crucial to specify the methods used to measure and control these variables during analysis to guarantee the reliability of the study outcomes.
2. Vitamin D and Seasonality:
    - Since vitamin D levels fluctuate considerably with seasons and sunlight exposure, it is crucial to determine whether participant recruitment was evenly distributed throughout different seasons or if this variability might introduce bias into the study.
3. Laboratory Methods**:**
    - While PCR-RFLP is a viable method for analyzing the VDR FokI polymorphism, it may be less precise than sequencing or TaqMan assays. Please explain why this approach was chosen, considering factors such as cost, feasibility, and resource availability in local laboratories.
4. Sample Size Assumptions:
    - The assumed odds ratio for the FokI polymorphism effect (around 3.9) might be an overly optimistic estimate. Performing a sensitivity analysis with different effect sizes could support the justification for the chosen sample size.
5. Ulcer Documentation:
    - Using PUSH scoring and photography for ulcer documentation is suitable; however,

it's important to specify measures like staff training, double scoring, or adjudication to minimize inter-observer variability.

6. Ethical Safeguards:
    - Since the study involves collecting patient photographs, it is essential to outline the methods for anonymization, secure storage, and obtaining informed consent for possible publication, ensuring compliance with ethical research standards.

Minor Points

- Include the total planned sample size in the abstract.
- Add an exploratory objective concerning potential gene–environment interaction (vitamin D and VDR polymorphism).
- Explain how missing data will be managed.
- Incorporate a flow diagram of participant recruitment to enhance clarity.
- Clearly specify which covariates will be adjusted for in the multivariate analysis.
- Indicate if correction for multiple testing will be used for genetic model comparisons.
- Clearly state limitations, such as the single-centre design, the inability of a case-control study to establish causality, and the potential for residual confounding.

These points aim to enhance the clarity, reliability, and ethical standards of the study protocol, ensuring it makes a substantial contribution to the field.

- Are sufficient details of the methods provided to allow replication by others?
  Partly – The methods are thorough overall, but some areas need more clarification to ensure full reproducibility. These include details on controlling confounders, inter-observer reliability in ulcer grading, the rationale for choosing PCR-RFLP over other genotyping techniques, and how seasonal variation in vitamin D was managed.
- Are the datasets clearly presented in a useable and accessible format?
   Not applicable – This is a study protocol; no datasets are presented yet.

Is the rationale for, and objectives of, the study clearly described?

Yes

Is the study design appropriate for the research question?

Yes

Are sufficient details of the methods provided to allow replication by others?

Partly

Are the datasets clearly presented in a useable and accessible format?

Not applicable

Competing Interests: No competing interests were disclosed.

Reviewer Expertise: Dr. Ravindra P. Turankar is a researcher in leprosy and neglected tropical diseases, with expertise in Mycobacterium leprae transmission, host susceptibility, and environmental surveillance. His work spans molecular diagnostics, CRISPR-based tools, cytokine

profiling, and One Health approaches. He emphasizes community engagement, WASH interventions, and AI-powered surveillance to advance early diagnosis, disability prevention, and climate-resilient strategies for leprosy elimination.

I confirm that I have read this submission and believe that I have an appropriate level of expertise to confirm that it is of an acceptable scientific standard, however I have significant reservations, as outlined above.

Author Response 15 Oct 2025

P. Anthony Halim

We thank the reviewer for the thoughtful and encouraging comments regarding the significance and methodological design of our study. Below, we respond to each of the points raised.

Major Comments

1. Confounders:

Reviewer comment: "The multifaceted factors leading to ulceration in leprosy encompass neuropathy, deformities, nutrition, footwear, and sun exposure. It is crucial to specify the methods used to measure and control these variables during analysis to guarantee the reliability of the study outcomes."

Response: We have expanded the "Procedures and examinations" section to provide detailed descriptions of how each of the relevant factors is assessed, including structured history taking, Semmes-Weinstein monofilament testing for neuropathy, physical examination for deformities, BMI classification for nutritional status, and sun exposure estimation. Footwear habits are not formally assessed, but are indirectly represented by socioeconomic status and presence of deformities. Given that this study is conducted in an urban setting, regular footwear use is generally expected among participants. These variables will also be incorporated into bivariate and multivariate analyses as described in the "Data analysis" section.

2. Vitamin D and Seasonality:

Reviewer comment: "Since vitamin D levels fluctuate considerably with seasons and sunlight exposure, it is crucial to determine whether participant recruitment was evenly distributed throughout different seasons or if this variability might introduce bias into the study."

Response: We acknowledge the important influence of seasonality on vitamin D levels. To minimize this variability, participant recruitment was planned and completed during the Indonesian dry season (March to September 2024), which provides stable sun exposure. This has been explicitly stated in the Abstract and the "Subject recruitment" section.

3. Laboratory Methods (PCR-RFLP vs. Other Genotyping Methods):

Reviewer comment: **"**While PCR-RFLP is a viable method for analyzing the *VDR FokI* polymorphism, it may be less precise than sequencing or TaqMan assays. Please explain why this approach was chosen, considering factors such as cost, feasibility, and resource availability in local laboratories."

Response: We agree with the reviewer that PCR-RFLP may offer lower precision than newer

genotyping techniques. However, the *FokI* polymorphism is a well-characterized variant with a simple restriction site, and PCR-RFLP remains a well-accepted method in these settings. As we are analyzing only one SNP, the higher-throughput capacity of TaqMan or next-generation sequencing is not required for this study. Additionally, PCR-RFLP was chosen based on cost-effectiveness and existing laboratory capacity. This rationale has been included in the “Discussion” section.

4. Sample Size Assumptions:
Reviewer comment: “The assumed odds ratio for the *FokI* polymorphism effect (around 3.9) might be an overly optimistic estimate. Performing a sensitivity analysis with different effect sizes could support the justification for the chosen sample size.”
Response: We acknowledge that the assumed odds ratio of 3.9 for *FokI* polymorphism may be optimistic, as it is based on preliminary data in a different leprosy population. The “ Sample size*”* section now includes a statement acknowledging this assumption and its limitations. A note has also been added to the “Discussion” section to encourage cautious interpretation and future validation in larger studies.

5. Ulcer Documentation and Inter-Rater Reliability:
Reviewer comment: *“*Using PUSH scoring and photography for ulcer documentation is suitable; however, it's important to specify measures like staff training, double scoring, or adjudication to minimize inter-observer variability.”
Response: All ulcer scoring is conducted by the first author (a senior dermatology resident), directly supervised by two consultant dermatologists to ensure consistency. The use of the validated PUSH tool, as well as ulcer photography for retrospective verification, has been clarified in the “Procedures and examinations” section.

6. Ethical Safeguards for Photographs:
Reviewer comment: “Since the study involves collecting patient photographs, it is essential to outline the methods for anonymization, secure storage, and obtaining informed consent for possible publication, ensuring compliance with ethical research standards.”
Response: Thank you for highlighting this. Clinical photographs are taken only following written consent. Photographs primarily involve distal extremities where the ulcers are located, which limit the likelihood of revealing identifiable features. In cases where identifying marks such as tattoos or moles are visible, they will be digitally blurred. All images are stored in an encrypted, password-protected USB flash drive accessible only to the study investigators. These details have been added to the “Ethics and Consent” section.

Minor Points

- Planned sample size added to Abstract.
- Exploratory gene-environment interaction (vitamin D and *FokI* genotype) is now mentioned as an exploratory analysis in the *Objectives* section.
- Missing data:
  As this is a prospective study, we anticipate complete data collection. However, if missing data arise, complete-case analysis or multiple imputation will be used if necessary. This has been added to the “Data Analysis” section.
- Flow diagram of participant recruitment will be developed during study conduct and

included in the final results publication. This has now been mentioned in the manuscript.
- Specify covariates for multivariate analysis:
  All collected sociodemographic and clinical variables may be included in the multivariate model if their bivariate p-value is <0.25, as outlined in the “Data Analysis” section.
- Correction for multiple testing in genetic model comparisons:
  As only one SNP (*FokI*) is analyzed, correction for multiple testing is not deemed necessary, as now clarified in the “Data analysis” section
- State limitations (single-centre, causality, residual confounding):
  Limitations regarding the single-centre setting, observational design, potential residual confounding, and potentially optimistic effect size for FokI polymorphism analysis have been added to the Discussion section.

Competing Interests: None.

Version 1

Reviewer Report 29 July 2025

https://doi.org/10.5256/f1000research.173385.r392598

© **2025 Djawad K.** This is an open access peer review report distributed under the terms of the Creative Commons Attribution License, which permits unrestricted use, distribution, and reproduction in any medium, provided the original work is properly cited.

?

Khairuddin Djawad
[1] dermatovenereologi, Hasanuddin University (Ringgold ID: 64739), Makassar, South Sulawesi, Indonesia
[2] Wahidin Sudirohusodo General Hospital, Makassar, Indonesia
[3] dermatovenereologi, Hasanuddin University (Ringgold ID: 64739), Makassar, South Sulawesi, Indonesia
[4] Wahidin Sudirohusodo General Hospital, Makassar, Indonesia
[5] dermatovenereologi, Hasanuddin University (Ringgold ID: 64739), Makassar, South Sulawesi, Indonesia
[6] Wahidin Sudirohusodo General Hospital, Makassar, Indonesia

This research has novel value regarding the polymorphism of the *fokI* gene in ulcers and non-ulcers in leprosy sufferers, but it is necessary to exclude possible confounding factors such as the possibility of other systemic diseases accompanying leprosy such as osteophorosis, cardiovascular disease, other chronic infectious diseases, etc. Information is also needed on what tests you use to exclude the disease. This examination needs to be disclosed so that this research can be repeated

by other researchers. How do you ensure that the wound does not pass through the subcutis? What tests do you use? However, what needs to be taken into account is that wounds in leprosy patients are closely related to factors such as the severity of the leprosy, the patient's environment and the patient's lifestyle which have not been taken into account in this research protocol.

Is the rationale for, and objectives of, the study clearly described?
Yes

Is the study design appropriate for the research question?
Yes

Are sufficient details of the methods provided to allow replication by others?
Yes

Are the datasets clearly presented in a useable and accessible format?
Yes

Competing Interests: No competing interests were disclosed.

Reviewer Expertise: dermatologyst

I confirm that I have read this submission and believe that I have an appropriate level of expertise to confirm that it is of an acceptable scientific standard, however I have significant reservations, as outlined above.

Author Response 01 Sep 2025
P. Anthony Halim

We thank the reviewer for the constructive feedback on our work. Below, we provide point-by-point responses to each of the concerns raised.

1. Regarding potential confounders and inclusion/exclusion criteria:
Thank you for your thoughtful comment. We agree that addressing potential confounding factors is critical to ensure internal validity. In designing the exclusion criteria (Table 2), we focused on systemic conditions that are known to significantly alter vitamin D metabolism or immune function, including diabetes mellitus, end-stage kidney disease, severe liver dysfunction, malignancy, autoimmune disorders, and systemic infections. To operationalize this, we screen participants using a combination of structured clinical interviews and medical record reviews, including relevant laboratory and radiologic findings when available. We have clarified these procedures in the revised manuscript ("Case and control definitions" section).

2. Regarding methods to ensure wound depth:
We appreciate this important point. As stated in the inclusion criteria, eligible wounds must not extend beyond the subcutaneous layer. Wound depth is primarily assessed through clinical inspection of the ulcer base. In cases where slough or necrotic tissue obscures the

wound base, we would perform sharp debridement as part of standard clinical practice to allow proper visualization. Patients are eligible for inclusion if, following debridement, the wound bed is confined to the subcutaneous layer. Any wound showing evidence of deeper tissue involvement, such as fascia, tendon, or bone exposure, would be excluded. This has been clarified in the revised manuscript ("Procedures and examinations" section) to support reproducibility.

3. Regarding other factors that might influence the presence of trophic ulceration:
We thank the reviewer for this important observation. We fully agree that trophic ulceration in leprosy is multifactorial. In this study, we collect detailed sociodemographic data (including occupation, education level, and household income) as well as clinical variables (nutritional status, smoking history, disease duration, WHO classification, Ridley-Jopling classification, history of leprosy reactions, presence of peripheral neuropathy, and other deformities). These variables serve as proxies for disease severity, environmental conditions, and lifestyle-related factors. All these factors are incorporated into bivariate and multivariate analyses to assess their potential as confounding factors. We have clarified this approach in both the "Procedures and Examinations" and "Data Analysis".

Competing Interests: No competing interests were disclosed.

The benefits of publishing with F1000Research:

- Your article is published within days, with no editorial bias
- You can publish traditional articles, null/negative results, case reports, data notes and more
- The peer review process is transparent and collaborative
- Your article is indexed in PubMed after passing peer review
- Dedicated customer support at every stage

For pre-submission enquiries, contact research@f1000.com

F1000Research
