# Supplementary material for: Comparison of serum vitamin D level and vitamin D receptor gene FokI polymorphism in leprosy patients with and without trophic ulcers: A case-control study
Source: PLoS Negl Trop Dis. 2026 Apr 10;20(4):e0014205. doi: 10.1371/journal.pntd.0014205 (PMC13086432; doi:10.1371/journal.pntd.0014205)
Supplement: Attachment — Submitted filename: Response to reviewers_PLOS NTD.pdf [file pntd.0014205.s005.pdf]

Reply to reviewers: PNTD-D-26-00033

Comparison of serum vitamin D level and vitamin D receptor gene *FokI* polymorphism in leprosy patients with and without trophic ulcers: A case-control study

**Comments**	**Author Response**
**Journal Requirements**	
1) Please ensure that the CRediT author contributions listed for every co-author are completed accurately and in full.<br>At this stage, the following Authors/Authors require contributions: Paulus Anthony Halim, Sondang P. Sirait, Eliza Miranda, Yulia Ariani, Wresti Indriatmi, and Hok Bing Thio. Please ensure that the full contributions of each author are acknowledged in the "Add/Edit/Remove Authors" section of our submission form.	We thank the Editors for this reminder. We have now completed the full CRediT author contributions for all listed authors in the submission form.
2) We ask that a manuscript source file is provided at Revision. Please upload your manuscript file as a .doc, .docx, .rtf or .tex. If you are providing a .tex file, please upload it under the item type u2018LaTeX Source Fileu2019 and leave your .pdf version as the item type u2018Manuscriptu2019.	We thank the journal for this instruction. The revised manuscript source file (.docx) has been uploaded as requested.
3) Please upload all main figures as separate Figure files in .tif or .eps format. For more information about how to convert and format your figure files please see our guidelines:<br>https://journals.plos.org/plosntds/s/figures	Thank you for this reminder. All figures have been processed using the NAAS tool and uploaded as separate files in the required format (.tif) according to the journal guidelines.
4) Please amend your detailed Financial Disclosure statement. This is published with the article. It must therefore be completed in full sentences and contain the exact wording you wish to be published.	We thank the Editors for this reminder. The Financial Disclosure statement has been revised to comply with journal requirements and now contains the exact format intended for publication.
**Methods**	-Are the objectives of the study clearly articulated with a clear testable hypothesis stated?<br>-Is the study design appropriate to address the stated objectives?<br>-Is the population clearly described and appropriate for the hypothesis being tested?<br>-Is the sample size sufficient to ensure adequate power to address the hypothesis being tested?<br>-Were correct statistical analysis used to support conclusions?<br>-Are there concerns about ethical or regulatory requirements being met?
Reviewer #1: The purpose of this study is clearly explained.<br>The research design used in this study aligns with the objectives to be achieved.<br>The proposed hypothesis is clear and in accordance with the hypothesis proposed by the researcher.<br>The sample size for a case-control design between patients with and without the disease is appropriate and	We thank the reviewer for this encouraging comment.

sufficient to ensure power and answer the hypothesis being tested.<br>The statistical analysis used reflects the overall conclusions.<br>There are no ethical issues in this research and it complies with applicable regulations.	
Reviewer #2: All questions are addressed in full	We thank the reviewer for this encouraging remark.
Reviewer #2 (PDF file): Did the questionnaire include dietary questions, related to sources related to vit D?	We thank the reviewer for this thoughtful question. Detailed dietary vitamin D intake was not assessed, as recall-based dietary reporting in this population may be unreliable and sun exposure represents the predominant source of circulating vitamin D. We have clarified this briefly in the Methods section.
Reviewer #2 (PDF file): Although numbers are sufficient, would higher numbers have given you even stronger results?	We appreciate this comment. The sample size was determined a priori based on the primary objectives. While a larger cohort would likely improve the precision of effect estimates, particularly for genetic models, the current sample size was sufficient to address the predefined study objectives. This has been clarified further in the Strengths and Limitations subsection.
Reviewer #3: The objectives and hypothesis are stated clearly. Power analysis 80% power and 5% significance performed and sufficient for the study. Approved by the Ethics Committee of Universitas Indonesia (No. KET-82/UN2.F1/ETIK/PPM.00.02/2024).<br><br>No major new analyses required.	We thank the reviewer for this positive assessment.
Reviewer #4: The objective and study design are well explained, and the manuscript is written in a standard format with details.<br>The only concern I have is the prevalence of Vit D level in general population in Indonesia. I request the researchers to add the prevalence of Vit D level in non-leprosy population and then compare with leprosy population. It seems that Vit D level is low in general population in most of the south Asian countries.	We thank the reviewer for this important suggestion. We have added additional information regarding the prevalence of vitamin D deficiency in the general Indonesian and Southeast Asian populations in the Introduction and Discussion sections. This allows clearer comparison between vitamin D status in non-leprosy populations and our study cohort.
Reviewer #5: - The sample size appears adequate to detect differences in serum vitamin D levels between groups. However, it may be insufficient for robust genetic association analyses, as indicated by wide confidence intervals and low genotype frequencies. The absence of a formal power calculation for the genetic component limits inference, and these findings should be interpreted as exploratory.	We thank the reviewer for this important observation. We agree that the modest sample size may limit the precision of the genetic association estimates, as reflected by the wide confidence intervals. We have revised the Discussion and Limitations sections to clarify that the genetic findings, particularly those related to inheritance model analyses, should be interpreted cautiously and considered exploratory.
Reviewer #5: - Several genetic comparisons were conducted, including genotype, allele, and inheritance model analyses. Although the findings are biologically plausible, the lack of adjustment for multiple testing (e.g., Bonferroni or false discovery rate) should be acknowledged, as it may increase the risk of false-	We thank the reviewer for this thoughtful comment. In this study, only a single pre-specified candidate polymorphism (*VDR FokI*) was evaluated based on prior biological rationale; therefore, formal correction for multiple testing was not applied.

positive associations. To strengthen statistical rigor, the authors may consider applying a correction for multiple genetic comparisons (e.g., false discovery rate) or, alternatively, explicitly state that the genetic analyses are exploratory in nature.	Nevertheless, we agree that exploring several inheritance models may increase the risk of type I error and thus warrants cautious interpretation. Accordingly, we have revised the Discussion to explicitly state that these analyses are exploratory in nature and that the findings should be interpreted with caution.
Reviewer #6: Study design and objective very clearly stated. Ethical approval is taken for the study. Conclusion derived from the study is acceptable.	We thank the reviewer for the positive evaluation of our study design and ethical conduct.
Reviewer #7: -Are the objectives of the study clearly articulated with a clear testable hypothesis stated? Yes<br>-Is the study design appropriate to address the stated objectives? Yes<br>-Is the population clearly described and appropriate for the hypothesis being tested? Yes<br>-Is the sample size sufficient to ensure adequate power to address the hypothesis being tested? Yes<br>-Were correct statistical analysis used to support conclusions? Yes<br>-Are there concerns about ethical or regulatory requirements being met? No	We thank the reviewer for the positive assessment of our work.
**Results**	-Does the analysis presented match the analysis plan?<br>-Are the results clearly and completely presented?<br>-Are the figures (Tables, Images) of sufficient quality for clarity?
Reviewer #1: done	We thank the reviewer for the positive assessment of our work.
Reviewer #2: This is a good and thorough study / analysis, and excellent way of narrowing down on vit.D mechanisms in TUs, with adequate attention for confounders.	We thank the reviewer for the encouraging comments.
Reviewer #2 (PDF file): This also means that vit.D suppelmentation is only one potential measure to be taken, the other variables can also be intervened with? Is this addressed in the discussion?”	Thank you for this important comment. We agree that trophic ulcer development is multifactorial. In accordance with our multivariable results, vitamin D status represents one potentially modifiable factor, whereas educational level may reflect broader social determinants of health in general. Non-ulcer deformities may be mitigated through improved prevention of disability (POD) care, although established deformities are often irreversible. We have clarified this perspective in the Discussion.
Reviewer #2 (PDF file): “As this was a case-control study, temporal direction cannot be determined, and the findings should not be interpreted as evidence of causality.” > True, but is causality established for diabetic ulcers?	We appreciate this important point. Even in diabetic ulcers, where the evidence is more robust, vitamin D is generally regarded as a contributory rather than a sole causal factor, given the multifactorial nature of chronic wound development. We have refined the Discussion to reflect this broader context.
Reviewer #3: Findings clear and well-structured.	We appreciate the reviewer’s positive assessment of the clarity and structure of our findings.
Reviewer #4: Yes, the results are presented very well and clearly.	We thank the reviewer for this remark.
Reviewer #5: - Results are clearly organized and logically presented, with appropriate reporting of effect estimates, confidence intervals, and p-values. The	We appreciate this important point. Additional clarification has been added to the Discussion to address the limited precision reflected by wide confidence intervals in the

distinction between unadjusted and adjusted analyses is clear. Minor clarification could improve interpretation, particularly regarding the clinical relevance of wide confidence intervals in genetic analyses and the limitations of correlation analyses.	genetic analyses and to more carefully interpret the clinical meaning of the observed correlation analyses.
Reviewer #6: Yes; result presented on the basis of data analysis is well presented.	We thank the reviewer for the positive assessment of our work.
Reviewer #7:<br>-Does the analysis presented match the analysis plan? The paper would be improved if the authors instead of showing the value of Serum 25(OH)D levels (line 179), include in the manuscript a scatter dot plot showing the serum 25(OH)D levels. This would allow the reader to better understand which patients are driven the difference in the Leprosy patients with TU. In addition, it would be interesting to associate the vitamin D values with the clinical forms of leprosy. Is there any difference, among leprosy with TU group, in the 25(OH)D levels between the patients with BT/BB and BL/LL? More specifically, are the vitamin D levels in BL/LL patients lower than in BT/BB patients?<br>-Are the results clearly and completely presented? Yes<br>-Are the figures (Tables, Images) of sufficient quality for clarity? Yes	We thank the reviewer for this insightful suggestion. In response, we have added a scatter dot plot showing the distribution of serum 25(OH)D levels between groups (new Fig 1). Based on the distribution of individual data points, we can observe that the difference is not driven by a small subgroup.<br><br>Additionally, we performed exploratory subgroup analyses according to Ridley-Jopling classification (BT/BB vs BL/LL). No significant differences in serum vitamin D levels were observed between these clinical forms in either the overall cohort or within TU and non-TU groups.<br><br>We also conducted subgroup correlation analyses between vitamin D levels and PUSH score. A moderate inverse correlation was observed in the BT/BB subgroup ($n = 18$; $r = –0.605$, $p = 0.008$), whereas no significant correlation was found in the BL/LL subgroup ($n = 23$; $r = –0.079$, $p = 0.719$). These findings have been added to the Results section and are discussed as exploratory observations.
**Conclusions**	-Are the conclusions supported by the data presented?<br>-Are the limitations of analysis clearly described?<br>-Do the authors discuss how these data can be helpful to advance our understanding of the topic under study?<br>-Is public health relevance addressed?
Reviewer #1: done	We thank the reviewer for this encouraging remark.
Reviewer #2: Fully correct!	We thank the reviewer for the positive assessment of our work.
Reviewer #2 (PDF file): Regarding further studies: I sincerely hope this is now planned for? If so, mention it here	We appreciate the reviewer's encouragement. While further prospective and interventional studies are warranted, no finalized protocol is currently in place. We have therefore retained a general recommendation for future research rather than specifying concrete plans.
Reviewer #3: Reverse causality might be worth mentioning, while remains unaddressed by authors, there is still a possibility that chronic inflammatory state and physical limitations that lead to reduced sun exposure, dictated by ulcers, might lead to a secondary depletion of Vitamin D.	We thank the reviewer for this important comment. We have expanded the Strengths and Limitations section to more explicitly acknowledge the possibility of reverse causality, including reduced mobility and decreased sun exposure related to chronic ulcers, as well as the potential influence of chronic inflammation on vitamin D metabolism.
Reviewer #3: The data support the conclusion that low levels of vitamin D and FokI polymorphism are independent factores associated with ulcers. The authors described the single-centre design and cross-	We thank the reviewer for this encouraging remark.

sectional nature, very relevant aspect.<br>Bringing the discussion of the role of Vitamin D in leprosy cases may be highly relevant for endemic regions that requires a low-cost intervention that may reduce morbidity of trophic ulcers.	
Reviewer #4: Yes, the conclusion is supported by the data presented.	We thank the reviewer for this encouraging remark.
Reviewer #5: - Key limitations related to the observational case–control design, potential reverse causality, and limited causal inference are acknowledged. However, additional limitations—particularly those related to genetic analyses (sample size, multiple comparisons, and population stratification)—could be more explicitly discussed to strengthen transparency.	We appreciate this suggestion and have revised the Limitations section to more explicitly discuss genetic analysis-related limitations, including sample size constraints. We have also clarified in the Statistical Analysis section that the inheritance model analyses were exploratory in nature. As ethnicity distribution was comparable between groups and broadly reflective of the Jakarta population, significant population stratification effects are unlikely.
Reviewer #6: Conclusion is clearly mentioned. Authors have suggested that will be helpful for understanding about tropic ulcer and further management , that may very important for patients suffering from ulcer	Thank you for the positive remarks.
Reviewer #7: -Are the conclusions supported by the data presented?<br>Yes, except for the graph that shows the correlation between 25(OH)D levels and PUSH scores. Although there is a significance, the correlation is extremely weak, which was recognised by the authors, and even with $p<0.05$ the correlation is not biologically relevant. Thus, it is not possible for the authors to claim that "a modest inverse correlation was also found between vitamin D levels and ulcer severity" (lines 227-228). Also, if the authors perform this correlation analysis separating BT/BB and BL/LL, does the correlation becomes stronger?<br><br>-Are the limitations of analysis clearly described? Yes<br>-Do the authors discuss how these data can be helpful to advance our understanding of the topic under study? Yes<br>-Is public health relevance addressed? Yes	We thank the reviewer for the careful interpretation remark and analysis suggestion. The conclusion wording has been revised to describe the observed association as weak and to avoid overinterpretation. Additional subgroup analyses (BT/BB vs BL/LL) on correlation have been added and are presented as exploratory findings. A moderate inverse correlation was observed in the BT/BB subgroup ($n = 18$; $r = -0.605$, $p = 0.008$), whereas no significant correlation was found in the BL/LL subgroup ($n = 23$; $r = -0.079$, $p = 0.719$). These findings have been added to the Results section and are discussed as exploratory observations.
**Summary and general comments**	
Reviewer #1: done	We thank the reviewer for the positive evaluation of our work.
Reviewer #2: No further comments, this is a well-conducted study!	We thank the reviewer for the encouraging remark on our work.
Reviewer #3: The study is relevant and well-designed. Also has novel data regarding vitamin D and leprosy. Mentioning that Sanger or NGS could not be performed due to cost is a great point, but also a weakness of the paper. It is still an acceptable choice on resource-limited settings.	We thank the reviewer for the positive evaluation of our work.

Other relevant aspects includes the low cost of Vitamin D supplements as well as the possibility of using FokI genotyping to identify patients and prioritize intensive care and nutritional support.<br>That may be the case in other countries that has high levels of leprosy and gives the rise of studies that focus on nutritional aspects of this disease.	
Reviewer #4: the manuscript is written very well with clear study design and interpretation of results. It can be accepted.	We thank the reviewer for the positive evaluation of our work.
Reviewer #5: - This study explores the association between vitamin D status, a functional VDR polymorphism, and trophic ulcers in patients with leprosy, focusing on a clinically relevant complication that has received limited attention.<br>Strengths of the study include the objective assessment of serum 25(OH)D levels, the use of multivariable logistic regression to account for key confounders, and the investigation of a biologically plausible genetic variant, with genotyping quality supported by Hardy–Weinberg equilibrium. The findings related to vitamin D are internally consistent and of potential clinical relevance.<br>The study is nonetheless subject to limitations inherent to its case–control design, including the possibility of residual confounding and limited causal inference. In addition, the sample size may restrict the robustness of the genetic analyses. The absence of formal correction for multiple genetic comparisons, along with the possibility of population stratification, suggests that the genetic findings should be interpreted with caution and viewed primarily as exploratory.<br>Overall, the manuscript contributes useful data to an underexplored area of leprosy research and, with appropriate acknowledgment of its limitations, represents a relevant addition to the neglected tropical diseases literature.	We thank the reviewer for this constructive assessment. In response, we have strengthened the Discussion and Limitations sections to more clearly acknowledge the inherent constraints of the case-control design and the limited precision of the genetic estimates. We have also clarified in both the Statistical Analysis and Discussion sections that the inheritance model analyses were exploratory in nature and should be interpreted cautiously. We believe these revisions improve the balance of the manuscript.
Reviewer #6: Overall all considerations regarding study is well stated.	We thank the reviewer for the positive evaluation of our work.
Reviewer #7: The authors investigated a subject that it is relevant for the public health but usually neglected. Interesting results were obtained and the experimental design was well articulated with the questions proposed. The association between trophic ulcer (TU) and vitamin D levels, and also FokI polymorphism showed that vitamin D levels is likely linked with the formation of TU. However, the authors only showed the mean values of Serum 25(OH)D levels (line 179). It is preferable to build a plot (scattered plot as already suggested) to know whether the levels of 25(OH)D was homogeneous in leprosy patients with ulcers or if there is a subgroup among leprosy patients with TU that drives the reduction of 25(OH)D.	We thank the reviewer for the constructive suggestions. In response, we have included a scatter dot plot of serum vitamin D levels and performed additional subgroup analyses as suggested (described in more detail above). These additions have strengthened the interpretability of the manuscript.
