# Supplementary material for: Comparison of serum vitamin D level and vitamin D receptor gene FokI polymorphism in leprosy patients with and without trophic ulcers: A case-control study
Source: PLoS Negl Trop Dis. 2026 Apr 10;20(4):e0014205. doi: 10.1371/journal.pntd.0014205 (PMC13086432; doi:10.1371/journal.pntd.0014205)
Supplement: Attachment — Submitted filename: Rev2_Response to reviewers_PLOS NTD.pdf [file pntd.0014205.s006.pdf]

Response to reviewers: PNTD-D-26-00033

Comparison of serum vitamin D level and vitamin D receptor gene *FokI* polymorphism in leprosy patients with and without trophic ulcers: A case-control study

Comments	Author Response
Reviewer #7<br><br>My final suggestion is to include in figure 3 the correlation graph for the BT/BB subgroup (Fig 3B), and for the BL/LL subgroup (Fig 3C) and highlight that the weak correlation found for leprosy patients with TU is not biologically relevant but if they look only BT/BB group then the correlation could be relevant. For some reason, in severe forms of leprosy this correlation is not significant. It is important also that they make a brief discussion about that. What is the authors hypothesis for no correlation in BL/LL individuals? After clarify those issues I aggree with the publication of the manuscript.	We thank the reviewer for this important suggestion. As recommended, Figure 3 has been revised to include subgroup correlation plots for BT/BB (Fig 3B) and BL/LL (Fig 3C). We have also refined the Discussion to highlight this observation and clarify that the stronger correlation observed in BT/BB patients may reflect relatively preserved cell-mediated immunity in the tuberculoid spectrum, whereas in BL/LL disease impaired cellular responses and greater disease burden may reduce the apparent contribution of vitamin D. Given the modest subgroup size and exploratory nature of this analysis, the subgroup findings are interpreted cautiously as hypothesis-generating.
